# Trimeric autotransporter adhesins driving chain-like adhesion diversify surface colonization strategies in Shiga toxin-producing *Escherichia coli*

**DOI:** 10.1038/s41467-026-76304-x

**Published:** 2026-08-04

**Authors:** Yuto Kotaka, Naoki A. Uemura, Tadayuki Iwase, Kenichi Lee, Nozomi Ishijima, Daisuke Nakane, Hirotaka Kobayashi, Michiyo Kataoka, Yukihiro Akeda, Sunao Iyoda

**Affiliations:** 1 Department of Biological Sciences, Graduate School of Science, Tokyo Metropolitan University, Tokyo, Japan; 2 Department of Bacteriology I, National Institute of Infectious Diseases, Japan Institute for Health Security, Tokyo, Japan; 3 Department of Engineering Science, The University of Electro-Communications, Tokyo, Japan; 4 Research Center for Medical Sciences, The Jikei University School of Medicine, Tokyo, Japan; 5 Molecular Diagnostics and Therapeutics, The Jikei University Graduate School of Medicine, Tokyo, Japan; 6 Department of Infectious Disease Pathology, National Institute of Infectious Diseases, Japan Institute for Health Security, Tokyo, Japan; 7 Research Institute for Microbial Diseases, The University of Osaka, Osaka, Japan; 8 Graduate School of Medicine, The University of Osaka, Osaka, Japan; 9 Chulalongkorn University, Bangkok, Thailand

**Keywords:** Bacterial genomics, Bacteriology, Genomics

## Abstract

Bacteria frequently colonize host and environmental surfaces under fluid flow. Chain-like adherence pattern (CLAP) is an EibG-mediated surface colonization phenotype of certain Shiga toxin-producing *Escherichia coli* (STEC) that lack the locus of enterocyte effacement (LEE). EibG, an immunoglobulin-binding trimeric autotransporter adhesin, drives CLAP, but the temporal dynamics and genetic diversity underlying chain formation remain unclear. Here, we use live-cell time-lapse imaging to show that chains arise from single cells that elongate and divide without separation. Under flow, chains resist detachment and undergo shear-dependent fragmentation at cell-cell junctions, releasing viable clonal units that disperse downstream. Comparative genomics reveals diversity among EibG-related adhesins and identifies distinct lineages, including chain-like adhesins (Cla) that mediate CLAP while lacking IgG binding. Screening of 1,354 genomes from England shows that *claB* is present in 95.6% of strains from major LEE-negative STEC serotypes, highlighting its epidemiological prevalence. Targeted mutagenesis demonstrates that chain formation and IgG binding are mediated by distinct structural domains, revealing the modular functional architecture of these adhesins. Furthermore, we show that EibG, ClaA, and ClaB confer robust resistance to complement-mediated killing. Collectively, these findings establish CLAP as a dynamic, surface-associated strategy of LEE-negative STEC and reveal diversification among adhesins that drive this behavior.

## Abstract (web summary)

In this study, the authors demonstrate that trimeric autotransporter adhesins drive bacterial chain-like adhesion, expanding the surface colonization strategies of Shiga toxin-producing Escherichia coli and promoting bacterial dispersal.

## Introduction

Shiga toxin-producing *Escherichia coli* (STEC) are major foodborne pathogens that cause severe disease outbreaks worldwide. Young children and older adults are at increased risk of developing severe diseases^1^. Clinical manifestations range from abdominal pain and bloody diarrhea to life-threatening complications such as hemolytic uremic syndrome (HUS) and encephalopathy^2,3^. Most clinical STEC isolates harbor the locus of enterocyte effacement (LEE), which encodes the adhesin Intimin, a type III protein secretion system, and multiple effector proteins required for virulence^4,5^.

Nevertheless, several large-scale outbreaks have been caused by LEE-negative STEC strains^6–8^. A notable example is the 2011 outbreak in Germany caused by the LEE-negative STEC O104:H4 strain, which resulted in an unusually high rate of HUS and substantial mortality^7^. Subsequent genomic analyses revealed that this strain was a hybrid pathogen in which an enteroaggregative *E. coli* (EAEC) lineage had acquired a Shiga toxin-encoding prophage^9^. Multiple STEC virulence factors, including Shiga toxin, enterohemolysin, and several adhesins, are encoded on mobile genetic elements (MGEs), such as bacteriophages and plasmids, enabling horizontal transfer among diarrheagenic *E. coli* (DEC)^10,11^. Consequently, the horizontal acquisition of MGEs can convert strongly adherent DEC lineages into highly virulent pathogens, as illustrated by the emergence of the O104:H4 strain^12,13^.

In Japan, O91 is one of the most frequently isolated LEE-negative STEC serogroups from clinical cases. A similar trend has also been reported in the United Kingdom and across the European Union^14,15^. Many O91:H14 isolates exhibit a distinctive chain-like adherence pattern (CLAP) mediated by EibG, an immunoglobulin-binding protein structurally related to YadA in *Yersinia enterocolitica*^16,17^. Notably, some strains forming CLAP (excluding O91:H14) possess canonical EAEC virulence markers, suggesting evolutionary and functional convergence with the EAEC lineage^18,19^.

EibG is a 508-amino acid trimeric autotransporter adhesin (TAA) that mediates immunoglobulin binding and CLAP^17^. Structural studies of the related protein EibD have revealed a lollipop-like structure consisting of a globular head domain and right- and/or left-handed coiled-coil stalk regions^20^. EibG binds immunoglobulins in a non-immune manner by interacting with the Fc fragment of IgG^17^. Structural and functional analyses of Eib family proteins, including EibD, have further mapped this immunoglobulin-binding activity to the coiled-coil stalk region^20^. EibG expression is highly condition-dependent: robust expression is observed during static culture, whereas only minimal expression levels are detected under shaking conditions^21^. EibG-expressing cells also exhibit pronounced autoaggregation and biofilm formation^22^. Recent domain deletion and chimeric analyses have demonstrated that EibG-mediated chain formation requires an intact head domain, as deletion of the entire head domain—rather than the N-terminal domain (NTD) or left-handed parallel β-roll (LPBR) alone—abolishes the chain-formation phenotype^23^. However, it remains unclear whether chain formation and IgG Fc-binding activity are functionally linked or are mediated by distinct structural domains of EibG.

Despite the clear importance of TAA-mediated chain formation in adherence and colonization, its contribution to STEC virulence is not fully understood. In this study, we performed comprehensive analyses combining time-lapse microscopy, comparative genomics of clinical isolates, and mutant characterization to elucidate the biological functions of EibG. We further identified and characterized Cla, a CLAP factor that lacks IgG Fc-binding activity, and assessed its epidemiological relevance by screening publicly available genomes from a UK surveillance cohort. These findings reveal that EibG-mediated chain formation represents a surface colonization strategy in STEC and provide insights into adhesive traits that may contribute to the emergence and pathogenic potential of hybrid DEC lineages.

## Results

### A single multicellular chain originates from a single cell

**Fig. 1:**
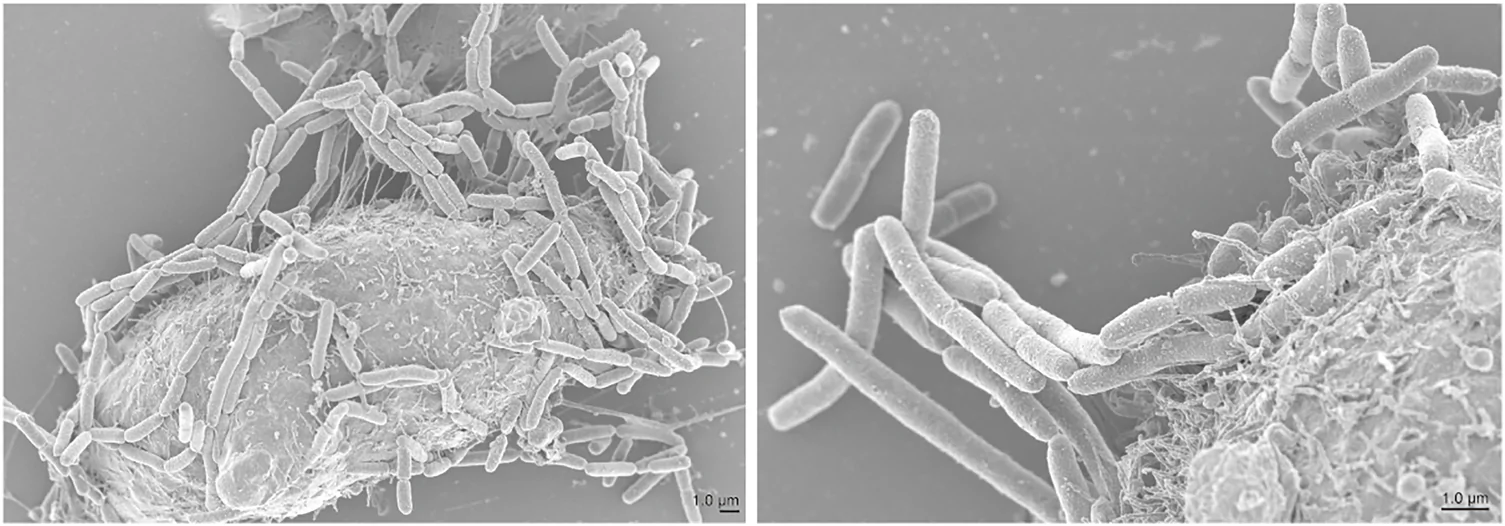
Scanning electron micrographs of HEp-2 cells infected with STEC strain JNEC-KY208. Cells were infected for 3 h under static conditions. Bacteria form multicellular chains that remain tightly attached to the host cell surface, displaying the characteristic chain-like adherence pattern (CLAP). Scale bar, 1 µm.

STEC O91:H14 strains (including O91:H–/Hg14) displayed a characteristic CLAP when infecting HEp-2 cells. To resolve the ultrastructure of this phenotype, we infected HEp-2 cells with strain JNEC-KY208 (O91:H–/Hg14, *stx1*^+^ *eibG*^+^) and examined the samples using scanning electron microscopy (SEM). SEM revealed that the bacteria remained firmly attached to the host cell surface, with pronounced constrictions at the interfaces between adjacent cells (Fig. 1).

**Fig. 2:**
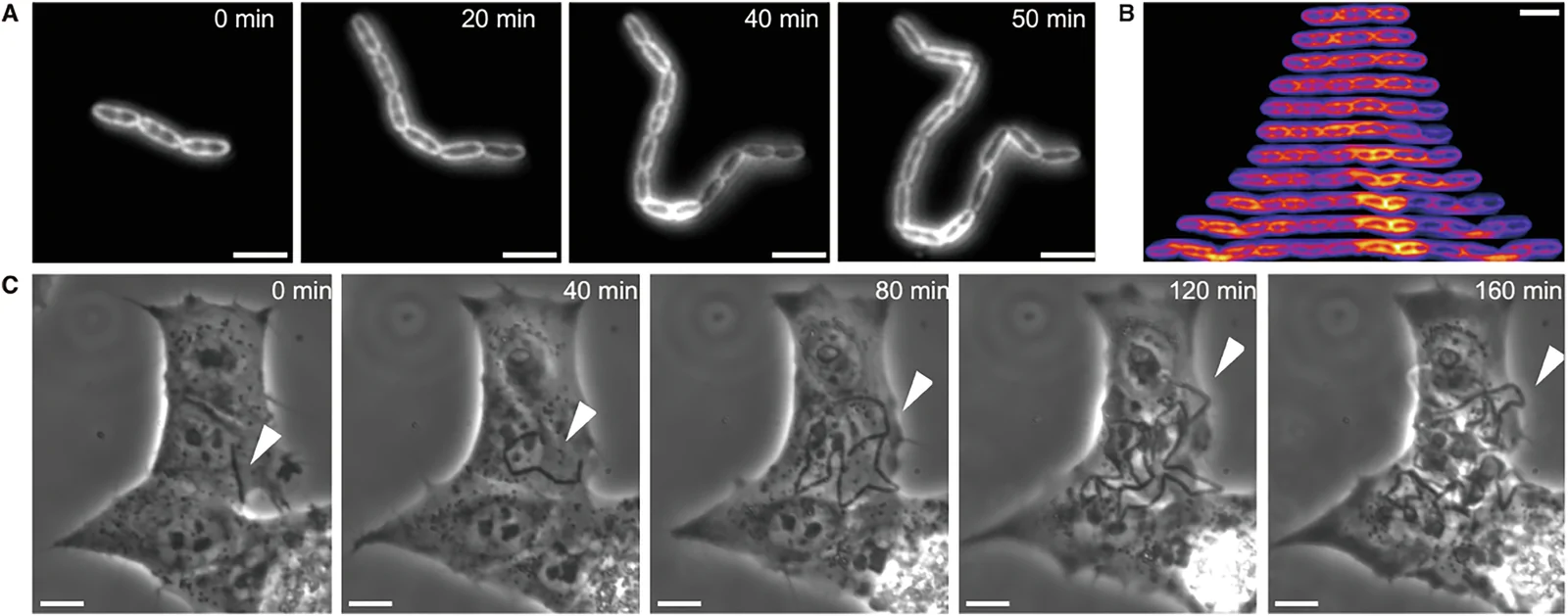
A single multicellular chain originates from a single cell in broth culture and during host-cell infection. **A** Time-lapse images of strain JNEC-KY208Δ*stx1* showing chain formation on a glass surface under dark-field microscopy at 37 °C. Scale bar, 5 µm. **B** Straightened time-lapse images at 5-min intervals. Cells were straightened from the images in (**A**) using image processing and are shown in pseudo color with adjusted contrast. Scale bar, 5 µm. **C** Time-lapse images of bacterial chain formation on HEp-2 cells under phase-contrast microscopy at 37 °C. Scale bar, 10 µm. The white arrowhead indicates bacteria attached to the cell surface.

Previous reports of CLAP have relied exclusively on static observations of infected HEp-2 cells at fixed time points and thus have not resolved how individual bacteria transition from single cells to multicellular chains. To visualize its dynamics, we performed time-lapse microscopy of JNEC-KY208Δ*stx1* under static growth conditions. Approximately 60 min of dark-field imaging at 37 °C showed that a single bacterial cell elongated and subsequently underwent cell division without separation, giving rise directly to a chain formation (Fig. 2A and Movie S1). Straightened image analysis demonstrated that constituent cells within a chain divided asynchronously, with an estimated doubling time of approximately ~20 min (Fig. 2B).

Next, we investigated whether chain formation occurred in the same manner during host infection, a condition under which CLAP is strongly manifested. To monitor the infection dynamics, we established a 160 min label-free phase-contrast time-lapse imaging system at 37 °C. HEp-2 cells grown on glass slides were infected with JNEC-KY208 at a multiplicity of infection (MOI) of 100, statically incubated for 1 h in 5% CO₂, and then washed to remove non-adherent bacteria. Imaging revealed that adherent bacteria elongated and formed chains on host cells in a manner similar to that observed in broth culture (Fig. 2C and Movie S2).

### EibG-mediated multicellular chains function as a surface colonization strategy under flow conditions

**Fig. 3:**
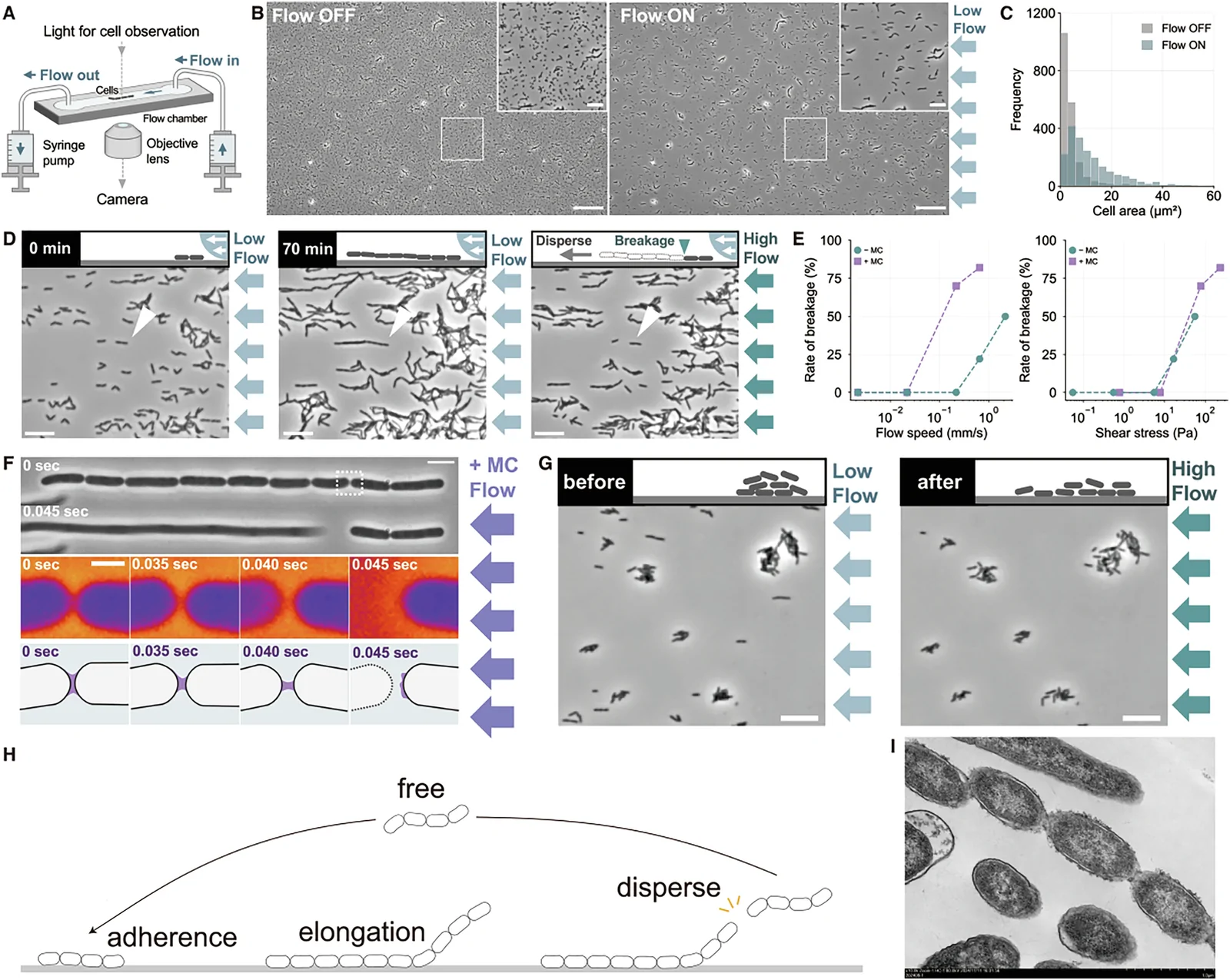
Multicellular chains function as a surface-colonization strategy under flow. **A** Schematic diagram of the experimental setup. The medium flow was applied from the right side of the fluid chamber using a syringe pump. Cell behavior was observed at room temperature. **B** Phase-contrast microscopy images of strain JNEC-KY208Δ*stx1* before and after applying a water flow of 22 µm/s for 10 min. The white square indicates the region enlarged in the insets. Scale bars, 100 µm for the main images and 20 µm for the insets. **C** Distribution of cell area shown in (**B**) (*N* = 2000 cells). Source data are provided as a Source Data file. **D** Chain formation and dispersion under water flow. Cells before applying a water flow (left), after applying a water flow of 22 µm/s for 70 min (center), and after applying a water flow of 22 mm/s for 1 min (right). The inset shows a schematic diagram of chain breakage and dispersion downstream. Scale bar, 20 µm. **E** Relationship between chain breakage and flow conditions. The ratio of broken cells among chain-forming cells was plotted against flow speed (left) and shear stress (right). Breakage during a 1-min flow of chain-forming clusters consisting of more than three connected cells was examined under conditions with or without 0.5% methylcellulose (MC) (*N* = 50 cells for each flow speed and shear stress). Source data are provided as a Source Data file. **F** High-speed imaging of chain breakage. Top: Phase-contrast microscopy images of chain breakage under a water flow of 2.2 mm/s with MC. Scale bar, 3 µm. The white square indicates the region enlarged in the middle panel. Middle: Enlarged time-lapse images of chain breakage shown in pseudo color. Scale bar, 0.5 µm. Bottom: Schematic images of chain breakage following the elongation of the intercellular distance. **G** JNEC-KY208Δ*eibG*/pGEM-*eibA* forming clump formation under water flow. Cells under a flow rate of 22 µm/s (left) and after applying a water flow of 22 mm/s for 1 min (right). The inset shows a schematic diagram of clump flattening. Scale bar, 20 µm. **H** Schematic model of the surface-colonization strategy inferred from microscopy. **I** Transmission electron micrographs of multicellular chains.

However, the adaptive role of CLAP under physiological and environmental flow conditions remains unclear. Previous studies have described characteristic bacterial dynamics under flow conditions that mimic natural and host-associated environments^24,25^. To assess how chain morphology responds to shear stress, we established a flow-chamber system coupled to a syringe pump that continuously supplied fresh medium at defined flow rates (Fig. 3A). Bacteria were introduced into the chamber and allowed to adhere to the glass surface during a 15 min incubation at room temperature. When medium was supplied at 0.1 µL/s for 10 min, single cells were almost swept away, whereas multicellular chains remained attached on the surface (Fig. 3B, C and Movie S3). This flow rate corresponds to a shear stress of 0.06 Pa and a near-surface flow speed of 22 µm/s (Fig. S1). These results indicate that chain morphology confers greater resistance to shear stress than the single-cell state.

To examine how longer chains respond to higher shear forces, bacteria were first grown under low-flow conditions (0.1 µL/s) within the chamber to allow formation of extended chains. Upon applying a flow rate of 100 µL/s, chains ruptured at intercellular constriction sites and released small fragments downstream (Fig. 3D and Movie S4). To test whether chain breakage depends on shear stress, we observed chain breakage under different viscosity conditions, with and without 0.5% methylcellulose (MC) (Fig. 3E and Movie S5). Chain breakage events were counted within 1 min at room temperature, under conditions that minimize the contribution of cell division. Even at the same flow speed, the presence of MC resulted in a higher rate of chain breakage, indicating that chain breakage depended on shear stress rather than flow rate. Notably, the force required to disrupt cell–cell junctions was lower than that required to detach the chains from the surface, indicating that fragmentation occurred prior to detachment (Movie S6). This provides an efficient means for disseminating clonal bacteria downstream. High-speed imaging was performed at the moment of chain breakage (Fig. 3F and Movie S7). The intercellular distance gradually increased and then suddenly ruptured at a certain point. These observations suggest that the breakage of chain-forming cells occurs due to the mechanical rupture of EibG-mediated connections at the poles of divided cells.

To clarify the advantages of CLAP under shear stress, we compared the adherence dynamics of EibG-mediated chains with those of EibA-mediated clumps using our flow assay system^23^. As expected, EibA-expressing cells formed robust aggregates that exhibited significantly higher attachment to the glass surface compared to adhered single cells (Fig. 3G and Movie S8). However, a fundamental mechanistic difference emerged under flow conditions. When exposed to high shear stress, EibA-mediated clumps underwent morphological flattening as unified structures but did not exhibit partial release. These findings demonstrate that, whereas clump formation is optimized for adherence, CLAP represents a specialized strategy to balances stable surface colonization with efficient downstream dispersal. This dual functionality may facilitate the rapid spread of STEC across host mucosal surfaces under physiological flow conditions.

Chain-mediated dispersal resembles recent observations in *Enterococcus*, where multicellular chains modulate colonization dynamics in response to flow^24^. Our findings demonstrate that Gram-negative STEC can exploit a similar architectural strategy: EibG-mediated adhesion promotes chain elongation, followed by shear-induced fragmentation, thereby expanding the spatial range of colonization (Fig. 3H). Transmission electron microscopy further confirmed complete cytoplasmic separation between adjacent cells, strongly suggesting that the released fragmented units are viable and capable of establishing new sites of colonization (Fig. 3I).

### EibG exhibits considerable sequence diversity

To investigate the conservation of the adhesins responsible for this phenotype, we chose 12 Japanese clinical STEC isolates as described in “Methods”. Together with JNEC-KY208, these isolates were subjected to whole-genome sequencing using the Illumina short-read and MinION long-read technologies. Hybrid assemblies generated using Hybracter yielded complete circular chromosomal contigs for all strains (Table 1). CheckM2 confirmed high-quality genomes, with completeness of 100% and contamination of ≤0.94%. In addition to Shiga toxin, various virulence factors were identified in these strains (Fig. S2).

**Table 1:** Complete genome sequences of 13 clinical STEC isolates determined in this study

Strain	Serotype	Genome size (bp)	Accession No.
JNEC-KY171	O146:H21	5,417,541	AP045167
JNEC-KY173	O75:H5	5,179,873	AP045169
JNEC-KY174	O128ab:H2	5,515,563	AP045170
JNEC-KY175	O8:H19	5,057,422	AP045171
JNEC-KY176	O146:H21	5,546,749	AP045172
JNEC-KY177	OgN4:H18	5,120,887	AP045173
JNEC-KY178	O23:H24	5,132,956	AP045174- AP045175
JNEC-KY179	O91:H14	5,054,153	AP045176
JNEC-KY180	O88:H25	5,100,032	AP045177
JNEC-KY202	O128ab:H2	5,431,201	AP045178
JNEC-KY203	O48:H45	5,041,191	AP045179
JNEC-KY204	O138:H48	5,299,796	AP045180
JNEC-KY208	O91:H14	5,024,628	AP045181

**Fig. 4:**
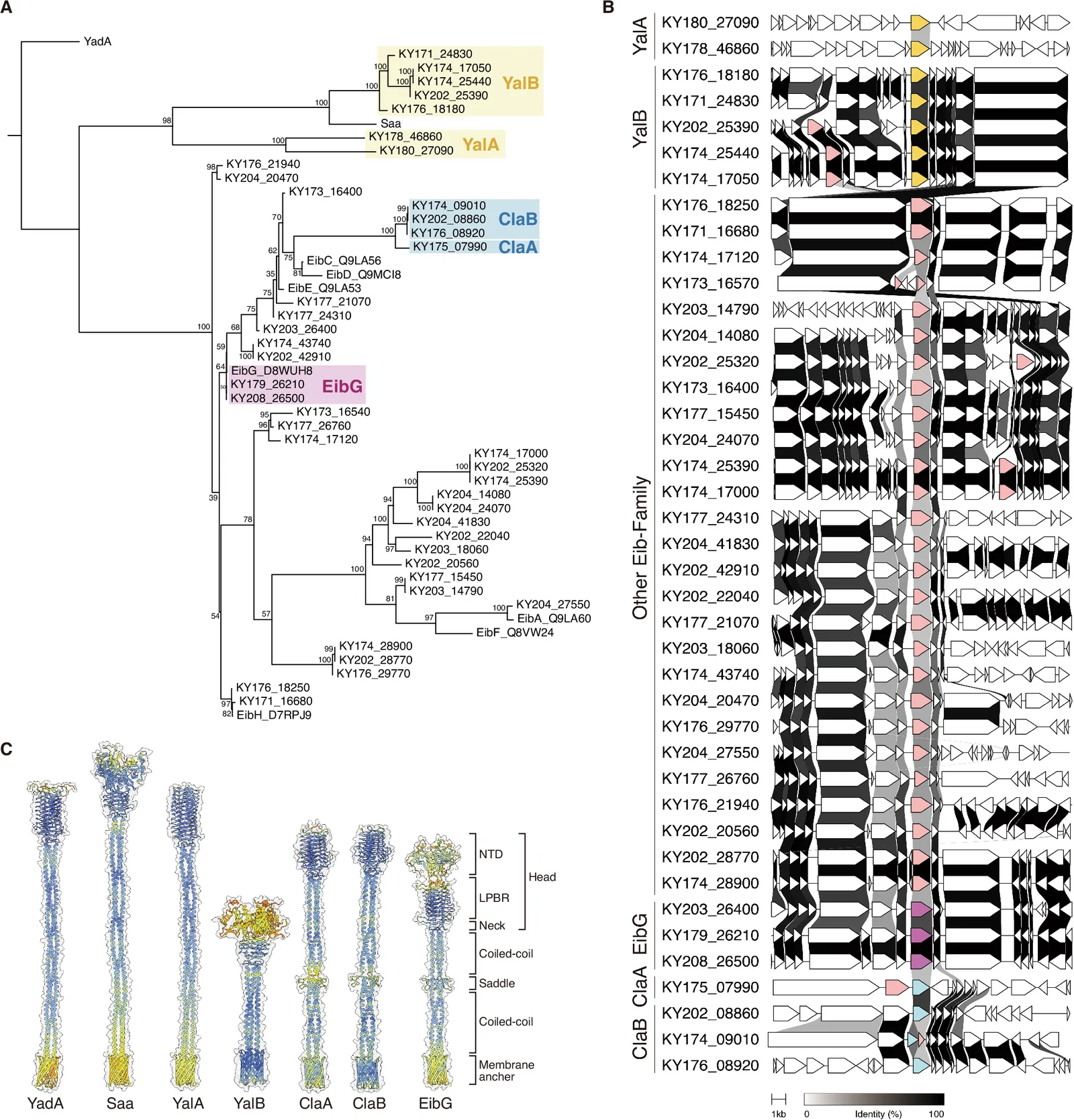
Phylogenetic, genomic, and structural analysis of EibG-like trimeric autotransporter adhesins. **A** Maximum-likelihood phylogeny of 41 EibG-like adhesins identified from 13 complete genomes of LEE-negative STEC and reference sequences. Signal peptides were removed by SignalP prior to alignment, and sequences were aligned using MAFFT and trimmed with trimAl. Tree inference was performed using IQ-TREE with ultrafast bootstrap support. Selected substitution model by ModelFinder is VT + F + G4. YadA was used as the outgroup to root the tree. The phylogeny includes two distinct adhesin groups identified in this study. These groups are indicated in the figure as Cla, shown in blue, and Yal, shown in yellow. **B** For each *eibG*-like gene, syntenic regions spanning 10 kb upstream and downstream are shown, visualized using clinker. The sequence homology is displayed in grayscale. **C** AlphaFold3 structural predictions of trimeric assemblies of representative adhesins. The sequence with the signal peptide removed by SignalP was used as input. The cartoon representation was color-coded according to the pLDDT score. NTD indicates the N-terminal domain, whereas LPBR indicates the left-handed parallel β-roll domain. Source data are provided as a Source Data file.

Using the 13 complete genomes as a database, we performed BLASTp searches with EibG (accession ADJ17706.1) as the query and identified 55 EibG-like amino acid sequences. After removal of signal peptides using SignalP, the sequences were aligned with MAFFT, trimmed with trimAl, and subjected to phylogenetic analysis using IQ-TREE3. Sequences lacking predicted signal peptides were excluded, resulting in 41 EibG-like proteins used for further analysis. The number of homologues per strain ranged from 1 to 8 copies, suggesting that gene duplication and subsequent diversification are ongoing processes within this adhesin family. Interestingly, several sequences clustered distantly from known Eib-family proteins and instead grouped more closely with Saa, an autoagglutinating adhesin of STEC (Fig. 4A). Because the functions of these proteins are unknown, we designated the two divergent clades as Yal (function unknown adhesin-like). To explore their potential evolutionary origins, we next analyzed their genomic contexts. Using clinker, we visualized loci spanning 10 kb upstream and downstream of each *eibG*-like gene. Known Eib-family proteins fell into three major genomic-context patterns, whereas the Cla (blue) and Yal (yellow) loci exhibited distinct, non-overlapping arrangements (Fig. 4B). While the majority of these homologues are encoded on the chromosome, we observed compelling evidence of genetic mobility. Specifically, the variant YalA was identified on both a plasmid (in strain JNEC-KY178) and the chromosome (in strain JNEC-KY180). This dual localization, together with the phylogenetic relationship between YalA and the plasmid-encoded adhesin Saa, strongly suggests a dynamic evolutionary history involving horizontal gene transfer and inter-replicon mobilization. Collectively, these results suggest that Cla and Yal are evolutionarily distinct from canonical Eib-family proteins and may represent separate adhesin lineages.

To compare structural similarities, we performed trimeric structural prediction using AlphaFold3, informed by the fact that TAA-family adhesins function as trimers. The Cla and Yal proteins displayed trimeric architectures that closely resembled the lollipop-like structures that are characteristic of YadA and EibG (Fig. 4C). Cla proteins exhibited a pronounced saddle domain, consistent with structural predictions for EibG. In contrast, the saddle domain was not predicted in YadA, Saa, or the Yal proteins. Furthermore, the NTD predicted for EibG was absent in both Cla and Yal, indicating that although these proteins share an overall trimeric structure, they differ in key structural features conserved among canonical Eib-family members.

### ClaA and ClaB are newly identified CLAP factors

**Fig. 5:**
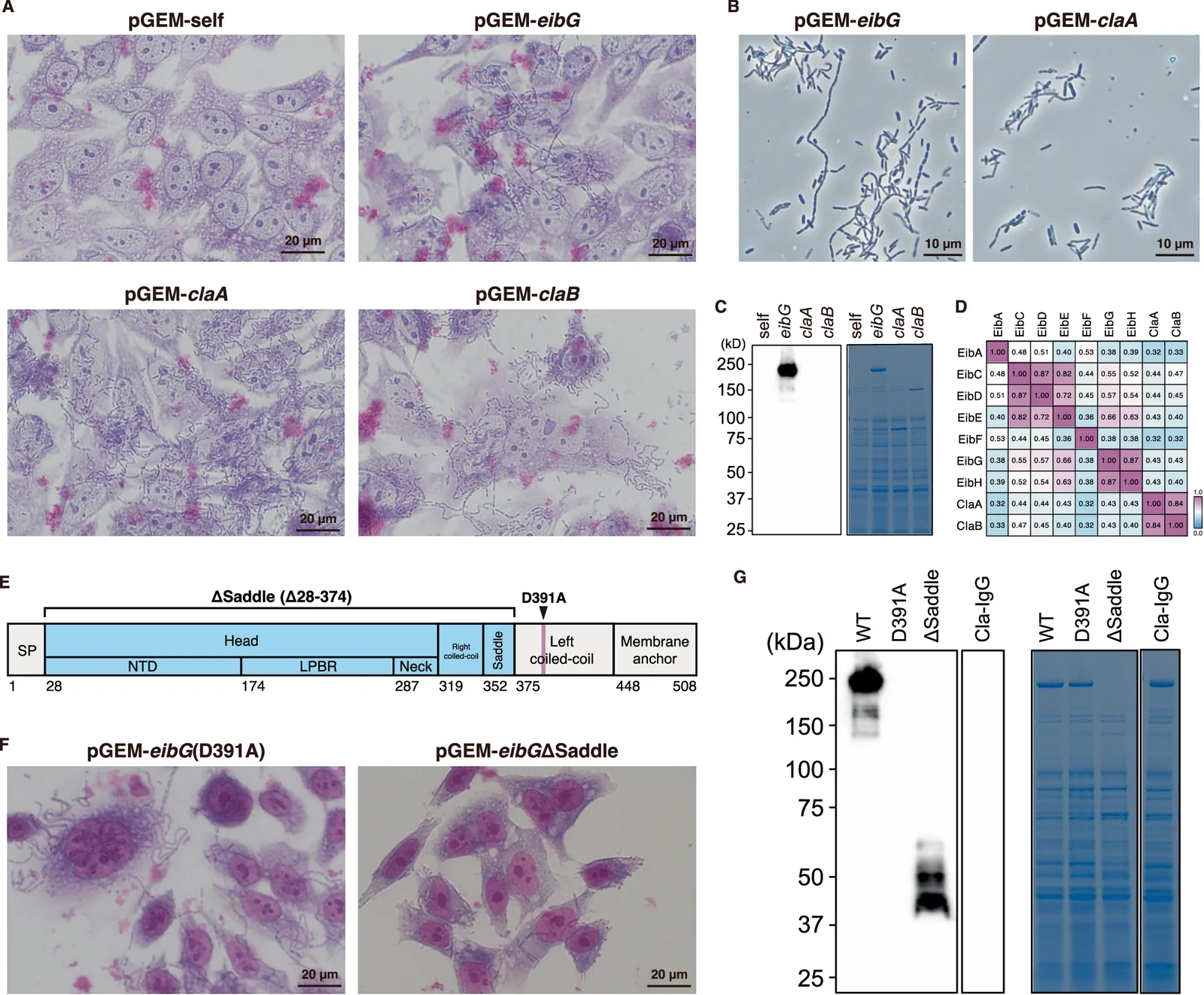
Functional characterization of Cla adhesins and structural separation of chain formation and IgG binding in EibG. **A** Complementation analysis of CLAP in the *eibG* deletion mutant by plasmid-borne *eibG*, *claA*, and *claB*. Image shows that deletion of *eibG* abolishes CLAP, which is restored by plasmid-borne expression of *eibG*. The JNEC-KY208Δ*eibG* strain was infected with HEp-2 cells using a strain in which *eibG*, *claA*, and *claB* were complemented from a pGEM plasmid and *eibG* upstream region (168 bp). After a 3 h incubation, the cells were fixed and stained with Giemsa. Scale bar, 20 µm. **B** JNEC-KY208 Δ*eibG* strain was cultured overnight under static conditions after introducing pGEM-*eibG* and pGEM-*claA*. Scale bar, 10 µm. **C** Immunoblotting assay of EibG, ClaA, and ClaB. Whole-cell lysates were separated by SDS-PAGE and probed with HRP-conjugated human IgG Fc (20 ng/mL) (left) or by total protein staining (right). High-molecular-weight band is detected only in strains expressing EibG. The label for each sample indicates the gene cloned into pGEM. Source data are provided as a Source Data file. **D** Sequence similarity matrix of Eib-family proteins and Cla adhesins. Pairwise amino acid sequence similarities were calculated using Clustal Omega and visualized as a heat map. Color scale indicates percent identity. **E** Domain architecture of EibG and positions of engineered mutations. The D391A substitution targets the residue aligned with the conserved IgG-binding aspartate in EibD. The Δsaddle mutant lacks the entire head–to–saddle region, which is indicated in the schematic by the solid line marking the deleted segment. SP indicates the Signal peptide, whereas NTD indicates the N-terminal domain and LPBR indicates the left-handed parallel β-roll domain. **F** Complementation analysis of CLAP in the *eibG* deletion mutant by plasmid-borne *eibG* (D391A), *eibG* (ΔSaddle). HEp-2 infection assays were performed as in (**A**). Scale bar, 20 µm. **G** Immunoblot and total-protein analysis of EibG mutants. Whole-cell lysates from strains expressing the indicated EibG variants were separated by SDS-PAGE and analyzed either by IgG Fc probing (left) or by total protein staining (right). The D391A mutant and Cla-IgG chimera mutant lacks detectable IgG Fc binding but retains the high-molecular-weight EibG band in the total-protein gel. In contrast, the Δsaddle mutant preserves IgG-binding activity, indicating that almost the entire passenger region is not required for Fc interaction. The labels for each sample indicate the *eibG* mutation cloned into pGEM, with WT representing wild-type EibG and Cla-IgG representing an EibG mutant with the IgG-binding site replaced by the Cla-type sequence. Source data are provided as a Source Data file.

To investigate the functional roles of ClaA and ClaB, we cloned each gene and expressed them in the JNEC-KY208 Δ*eibG* mutant. As expected, the Δ*eibG* strain exhibited a complete loss of CLAP, which was rescued by plasmid expression of EibG. Remarkably, expression of either ClaA or ClaB also restored CLAP (Fig. 5A). However, when bacteria were grown under static culture conditions without host cells, ClaA-expressing cells formed few if any chains, whereas EibG-expressing cells formed multicellular chains and exhibited autoaggregation (Fig. 5B).

Since Eib proteins exhibit IgG Fc-binding activity, we examined whether Cla proteins also possess this activity. For this experiment, whole-cell lysates were heated at 50 °C, separated by SDS-PAGE, transferred onto PVDF membranes, and probed directly with HRP-conjugated human IgG Fc fragment. In the strain transformed with pGEM-*eibG*, a prominent high molecular weight polypeptide corresponding to EibG was detected as an IgG Fc-binding band (Fig. 5C). In contrast, no IgG-binding signal was detected in cells expressing ClaA or ClaB (Fig. 5C). These results were further corroborated by the observation that the residues predicted to mediate IgG binding were not conserved in Cla (Fig. S3). Collectively, these findings indicate that ClaA and ClaB lack IgG Fc-binding activity, in contrast to EibG.

To determine whether ClaA and ClaB should be considered members of the Eib-family proteins, we evaluated their sequence relatedness using a similarity matrix generated by Clustal Omega. Eib-family proteins formed several similarity clusters (EibA; EibC–E; EibF; and EibG–H), consistent with their established phylogenetic relationships (Fig. 5D). In contrast, ClaA and ClaB did not cluster with any of these groups and shared only limited pairwise similarity with Eib proteins (<50%). Their distinct genomic contexts, lack of IgG Fc-binding activity, and limited similarity to canonical Eib proteins indicate that ClaA and ClaB form an adhesin lineage separate from the Eib family and are responsible for promoting CLAP. We therefore designate this lineage as chain-like adhesins (Cla).

To investigate whether *claA* and *claB* are present outside the Japanese clinical isolates analyzed in this study, we focused on a well-defined surveillance collection of LEE-negative STEC isolates from England^14^. In this cohort, serotypes O128:H2, O91:H14, and O146:H21 were among the top five STEC serotypes isolated from patients with gastrointestinal symptoms between 2014 and 2022. Using the accession numbers provided, we retrieved 1354 publicly available draft genomes that were publicly available and screened them using BLASTn, applying thresholds of >90% nucleotide identity and >90% query coverage to detect *eibG*, *claA*, and *claB*. Strikingly, *claB* was present in 97.82, 91.67, and 98.26% of isolates belonging to serotypes O128:H2, O91:H14, and O146:H21, respectively (Table 2).

**Table 2:** . Frequency of *claA*, *claB*, and *eibG*

	*claA*	*claB*	*eibG*
O128:H2	0/275(0.00%)	269/275(97.82%)	2/275(0.73%)
O91:H14	0/492(0.00%)	451/492(91.67%)	7/492(1.42%)
O146:H21	0/518(0.00%)	509/518(98.26%)	0/518(0.00%)
Other	0/69(0.00%)	65/69(94.20%)	0/69(0.00%)
Total	0/1354(0.00%)	1294/1354(95.57%)	9/1354(0.66%)

### Distinct structural domains mediate CLAP and IgG binding in EibG

Next, we examined the domain architecture underlying the dual functions of EibG: CLAP formation and IgG Fc-binding activity. Previous structural studies of EibD and multiple sequence alignments of Eib-family proteins have identified two residues (EibD^D378-Y388^) that are critical for interaction with the IgG Fc region, although their roles in CLAP have not been defined^20^. In addition, deletion of almost the entire passenger disrupts chain formation; however, whether this region contributes to IgG Fc-binding has remained unclear^23^.

To experimentally test the contribution of the predicted IgG Fc-binding site to CLAP, we mapped the corresponding residues identified in EibD^D378-Y388^ onto the EibG sequence. In EibG, the residue corresponding to EibD^Y388^ is already an alanine (EibG^A392^), whereas EibD^D378^ aligns with EibG^D391^. We therefore generated an EibG^D391A^ mutant to disrupt the conserved aspartate predicted to participate in IgG Fc binding activity (Fig. 5E). In parallel, we constructed a Δsaddle mutant lacking the almost the entire passenger (Fig. 5E). The EibG^D391A^ point mutant retained CLAP formation, and a high-molecular-weight EibG band was still detected by SDS–PAGE, but no IgG Fc-binding signal was observed (Fig. 5F, G). In contrast, the Δsaddle mutant lost CLAP formation while retaining IgG Fc-binding activity (Fig. 5F, G).

Consistent with these findings, the ClaA and ClaB mediated robust CLAP but lacked IgG Fc-binding activity (Fig. 5B, C), in line with the absence of the critical D391 residue in the Cla family. To validate this, we constructed a chimeric EibG mutant incorporating the ClaA-derived putative binding region (Fig. S4). Notably, while this chimeric mutant retained its ability to mediate CLAP, it failed to bind IgG Fc (Figs. 5G and S4). These results demonstrate that Cla proteins are functional variants where chain formation is preserved despite the loss of IgG-binding capacity. Our findings further indicate that the head-to-saddle domain and IgG Fc-binding residues contribute independently to EibG function, establishing that chain formation and IgG binding are mediated by distinct, non-overlapping structural determinants.

### TAA-mediated serum resistance and its contribution to systemic virulence

**Fig. 6:**
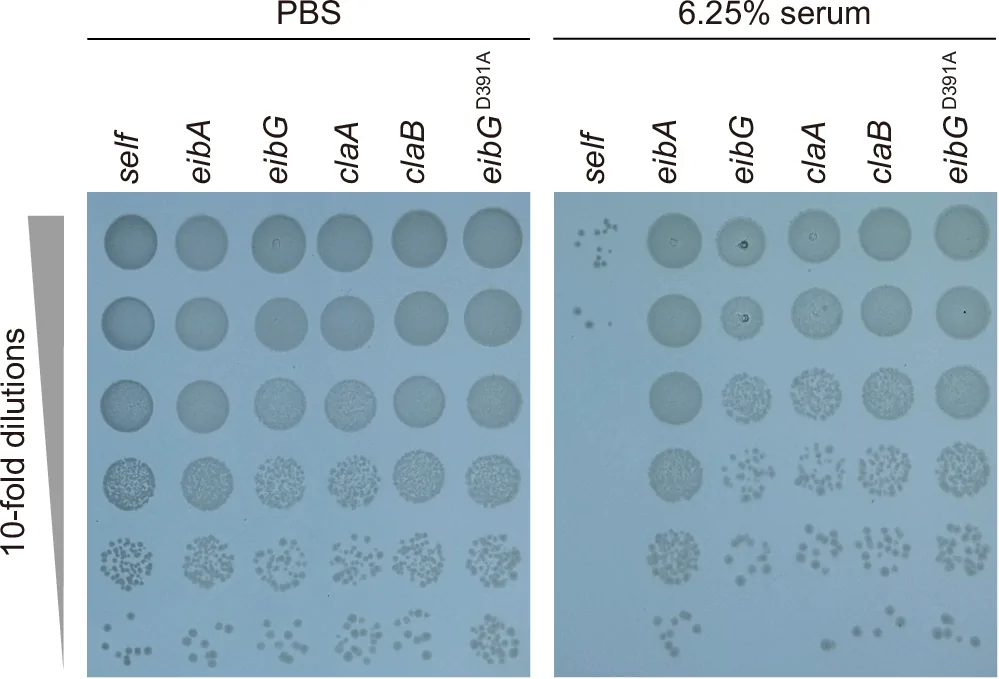
Cla proteins confer resistance to complement-mediated killing. **A** Overnight static cultures were harvested and washed with PBS. The bacterial cells were then resuspended and incubated in either 6.25% human serum or PBS as a negative control at 37 °C for 60 min with shaking. Following the incubation, tenfold serial dilutions were prepared and spotted onto LB agar plate supplemented with ampicillin. The label for each sample indicates the gene cloned into pGEM.

TAAs have previously been reported to contribute to evasion of complement-mediated killing^16^. Therefore, we investigated whether Cla confers serum resistance. Following the induction of TAA expression from plasmids in strain MG1655 under static culture conditions, the bacteria were suspended in either 6.25% human serum or PBS and incubated for 1 h. We found that both EibG and Cla conferred serum resistance to MG1655 (Fig. 6). Furthermore, serum resistance was maintained in EibG^D391A^, a mutant defective in IgG-binding activity (Fig. 6). These results indicate that TAA-mediated serum resistance is independent of non-immune binding to IgG.

We next investigated whether these functional characteristics of EibG contribute to bacterial virulence in vivo. Notably, Eib protein homologues can be found in some extraintestinal pathogenic *E*. *coli* (ExPEC) genomes (Fig. S5). In addition, they have been suspected of playing a role in virulence^26^. Given the multifaceted functions of EibG and its distribution among diverse pathogenic *E*. *coli* lineages, we employed an intraperitoneal (i.p.) infection model in specific-pathogen-free (SPF) mice to evaluate its pathogenic potential. This model provided a clear and reproducible means of evaluating the systemic impact of *E. coli* infection. Lethality provides a clear and quantifiable endpoint, allowing the evaluation of pathogenic consequences without the confounding variability inherent in gastrointestinal colonization models^27,28^.

Because Shiga toxin is the principal virulence determinant in STEC, we constructed a JNEC-KY208Δ*eibG* to allow assessment of EibG-specific effects. Mice were randomly assigned to receive 1.5 × 10⁸ CFU of either the Δ*stx1* parental strain or the isogenic Δ*stx1*Δ*eibG* mutant by intraperitoneal injection and were monitored for five days. Across three independent experiments (*n* = 31 for Δ*stx1*; *n* = 25 for Δ*stx1*Δ*eibG*), the Δ*eibG* mutant exhibited a significantly reduced lethality compared with the parental strain (*p* = 0.044) (Fig. S6). These findings demonstrate that EibG contributes significantly to STEC virulence in vivo.

## Discussion

In this study, we elucidated the genetic, structural, and functional basis of the CLAP observed in certain LEE-negative STEC. By integrating live-cell imaging, comparative genomics, and targeted mutagenesis, we demonstrated that EibG-driven multicellular chains constitute a surface colonization strategy for STEC. Our findings indicate that chain morphology enhances adhesive persistence under dynamic conditions, promotes clonal dispersal through shear-induced fragmentation, and contributes to pathogenic potential in vivo. Furthermore, the discovery of a new class of adhesins (Cla) that mediate CLAP but lack immunoglobulin-binding activity substantially broadens our current understanding of host-surface attachment mechanisms across DEC.

A central advancement of this study is the temporal resolution of CLAP development. Previous reports relied on static snapshots of infected cells and did not resolve whether CLAP arises through filamentous elongation, aggregation, or modified cell division. Our time-lapse analyses clearly show that chains originate from single progenitor cells that elongate and divide without separation. By integrating this imaging approach with microfluidic flow assays, we found that chain morphology provides an adaptive advantage under shear stress. Solitary cells detached readily under flow, whereas multicellular chains remained adherent and fragmented at intercellular constrictions, thereby releasing viable progeny downstream. This dispersion-driven mode of colonization parallels recent observations in *Enterococcus faecalis*, in which multicellular chains promote flow-dependent niche expansion^24^. Our results extend this paradigm to Gram-negative pathogens, suggesting that chain-based architectures represent a convergent strategy among host-associated bacteria that must maintain attachment and spread within fluid-exposed environments. Previous studies have identified chain formation as a key determinant of adhesion to plastic surfaces^23^. Our findings strongly support this model and indicate that chain formation, rather than clump formation, drives flow-dependent surface colonization. Furthermore, the ability of Eibs to adhere to a broad range of abiotic surfaces likely confers a selective advantage on STEC, promoting its survival and persistence across diverse environmental niches.

For DEC inhabiting the gastrointestinal tract, mucus flow and peristalsis generate substantial shear that can disrupt surface attachment^29,30^. Although higher shear forces promote more efficient chain fragmentation, our data indicate that chain breakage occurs at shear stresses of 0.78 Pa for observations more than 1 h (Movie S4). This value is broadly comparable to previously estimated shear stresses in the intestinal lumen and is therefore consistent with the possibility that such fragmentation may also occur in vivo^31^. Our results highlight a potential infection strategy that may be exploited by certain LEE-negative STEC in the intestinal environment. In this context, chain formation provides a significant advantage in terms of fitness. Accordingly, our findings add flow-dependent colonization to the repertoire of biological activities previously attributed to TAAs, which including adherence, biofilm formation, invasion, intracellular survival, serum resistance, and cytotoxicity.

Comparative genomic analysis revealed unexpected diversity within EibG-like adhesins. Although EibG and its homologues have historically been classified within the immunoglobulin-binding protein family, our phylogenetic analyses identified distinct clusters with unique genomic contexts, forming two lineages that we designate Cla and Yal. This diversification aligns with the known evolutionary plasticity of TAA-family proteins, which frequently undergo domain shuffling, repetition, and recombination, and are mobilized through horizontal gene transfer processes^32^. Structural predictions provide further evidence for the evolutionary divergence of Cla proteins from canonical Eib-family members. Cla proteins do not exhibit key features typically associated with the Eib family, such as conserved immunoglobulin G (IgG) Fc-binding residues and an NTD. This structural deviation suggests a functional specialization of Cla proteins in host-cell adherence rather than an interaction with immunoglobulins.

This functional divergence was corroborated by genetic analyses. Expression of ClaA or ClaB restored CLAP in the *eibG* deletion mutant, demonstrating that Cla proteins are sufficient to complement the loss of EibG with respect to this phenotype. However, under static culture conditions, Cla-expressing strains showed minimal chain formation, implying that Cla-dependent CLAP may be specifically displayed under host-associated or otherwise specialized environmental conditions. Notably, Cla proteins lack IgG Fc-binding activity, which directly establishes that CLAP and immunoglobulin binding are mechanistically separable phenotypes driven by distinct subsets of the TAA family proteins. A detailed examination of the domain architecture of EibG provided additional resolution for this functional separation. Substitution of the predicted IgG binding residue EibG^D391A^ abolished Fc recognition while leaving CLAP intact, whereas removal of the saddle domain eliminated CLAP while preserving IgG-binding. These results demonstrate that these two activities are encoded by distinct structural modules within EibG. These results highlight the modular organization of TAA adhesins and raise broader questions about the selective forces that preserve the immunoglobulin-binding ability within the Eib lineage. Furthermore, we found that serum resistance is conserved in Cla, suggesting that this protective phenotype represents a more generalized functional attribute across the TAA family, likely serving as a fundamental mechanism for host immune evasion.

Consistent with this hypothesis, ClaB was highly prevalent among clinical LEE-negative STEC isolates from England, particularly in serotypes O128:H2, O91:H14, and O146:H21, which were among the most frequently reported during 2014–2022. The high prevalence of ClaB in these clinically important lineages underscores its importance as an adhesin and suggests that Cla-mediated chain-like adhesion may contribute to their prevalence among clinical isolates. Our findings suggest that the chain-formation function of TAA proteins may provide a fitness advantage under specific ecological conditions.

Our in vivo experiments revealed that EibG contributes to pathogenicity. Although Shiga toxin remains the major driver of STEC-associated mortality, the *eibG* deletion mutant exhibited significantly reduced lethality even in a Δ*stx1* background. This indicates that EibG enhances disease severity through a toxin-independent mechanism. Potential explanations include improved colonization efficiency, increased persistence at host surfaces, or modulation of innate immune responses. While our current data do not resolve whether CLAP, IgG binding, or serum resistance phenotypes underlie the virulence effect, the domain analyses suggest that the physical properties of chain formation could themselves influence infection dynamics. Future studies employing domain-specific EibG mutants will be essential for dissecting host responses to these modular architectural features.

Collectively, our study expands the conceptual framework governing adhesion and virulence in STEC. Chain-like adherence, enabled by genetically diverse TAA family adhesins with distinct structural and functional properties, has emerged as a key strategy for surface colonization in certain LEE-negative STEC strains. Our identification of Cla proteins underscores the evolutionary capacity of DEC lineages to acquire and repurpose adhesion modules via horizontal gene transfer. A deeper understanding of the distribution, regulation, and host interactions of EibG, Cla, and related adhesins will be crucial for predicting the pathogenic potential of emerging LEE-negative STEC and hybrid DEC lineages. Moreover, these findings highlight TAA-mediated attachment as a promising target for therapeutic and preventive interventions.

## Methods

### Bacterial strains, media, primers, and plasmids

All resources used in this study are listed in Tables S1 (strains), S2 (primers), and S3 (plasmids). Bacteria were grown at 37 °C in LB Lennox medium (BD, USA) supplemented with ampicillin (100 μg/mL), chloramphenicol (20 μg/mL), and kanamycin (50 μg/mL).

### Genetic manipulation of STEC strains

Genetic manipulation of STEC strains was performed using the *λ* Red recombination method with the pSIM6 plasmid^33^. The *eibG* gene, including 168 bp of its upstream region, was cloned into pGEM-T Easy (Promega, USA). The pGEM-self was used as a negative control^34^. For *claA* and *claB* constructs, the *eibG* coding sequence (CDS) was replaced with each CDS via seamless cloning, preserving the *eibG* upstream region. The X-cocktail method, which relies on Exonuclease III (Takara Bio, Japan), was used as previously described^35^. Inverse PCR for site-directed mutagenesis was performed using partially overlapping primers and KOD One DNA polymerase (TOYOBO, Japan).

### Cell culture and infection with bacteria

HEp-2 cells were grown in Minimum Essential Medium (Sigma-Aldrich, USA) supplemented with FBS (10%) (Thermo Fisher Scientific, USA) at 37 °C in 5% CO_2_. For infection assay, HEp-2 cells were seeded at a density of 10^5^ cells/mL and cultured for 16 h. Prior to infection, the medium was replaced with FBS-free Minimum Essential Medium, followed by a 1-h incubation. The HEp-2 cells were then infected with the bacterial culture at a MOI of 100 and incubated for 3 h at 37 °C in 5% CO_2_. Following incubation, non-adherent bacteria were removed by washing three times with PBS. The infected cells were then fixed with methanol and subjected to Giemsa staining for visualization of the adherence patterns^17^.

### SEM

The samples were fixed overnight at 4 °C with a mixture of 2.5% glutaraldehyde, 2% paraformaldehyde, and 0.5% acetic acid. After fixation, the samples were dehydrated using a graded acetone series (50–99.5%) at room temperature and dried using a critical point dryer with CO₂ (CPD300, Leica Microsystems, Germany). The dried samples were coated with osmium using an osmium plasma coater (Neoc-Pro/P, Meiwafosis, Japan) and subsequently examined with a field-emission SEM (FE-SEM; SU8600, Hitachi High Technologies, Japan).

### Optical microscopy and data analyses

For time-lapse imaging of chain formation, bacteria and HEp-2 cells on the glass surface were visualized under an inverted microscope (IX83; Olympus, Japan) equipped with an ×40 objective lens (LUCPLFLN 40 × PH, N.A. 0.6), a CMOS camera (DMK33UX174; Imaging Source, Germany), and an optical table (ASD-1510T). Samples were visualized using a halogen lamp (U-LH100L-3; Olympus, Japan) through a bright-field condenser (IX2-LWUCD, N.A. 0.55; Olympus, Japan) for phase-contrast microscopy, or a dark-field condenser (U-DCD, NA0.8-0.92; Olympus, Japan) for dark-field microscopy. The microscope stage was heated using thermoplate (TP-110R-100; Tokai Hit, Japan). Time-lapse images were acquired every 10 s using IC Capture software (The Imaging Source) and converted into an AVI file without compression. Straighten image processing was performed by built-in function of Fiji software (ImageJ 1.53t).

For the flow experiment, the samples were examined under the inverted microscope equipped with ×10 and ×100 objective lenses (UPLFLN 10×2PH; N.A. 0.3, UPLXAPO100 × OPH, N.A. 1.45; Olympus, Japan), a CMOS camera, and an optical table. Time-lapse images were acquired using IC Capture software at time intervals of 0.005 s or 1 s and converted into an AVI file without compression. All data were analyzed by Fiji software. Images were thresholded with an intensity range of 0–102, and cell area was measured using the built-in Analyze Particles function without additional filtering for size or circularity. Chain breakage events were manually counted, defined as the separation of multicellular chains consisting of more than three connected cells into two or more fragments.

### Time-lapse imaging of chain formation

For the observation of bacterial chain formation on the glass surface, a tunnel chamber was assembled by taping coverslips with double-sided tape (~10 µm thick; 7070W, Teraoka, Japan), slightly modified from a previous report^25^. Bacterial suspensions in LB medium were introduced into the tunnel chamber. The chamber was incubated at 37 °C for 10 min on a stage equipped with a thermoplate, allowing cells to attach nonspecifically to the untreated glass surface. Unattached cells were washed away with LB medium, and the ends of the chamber were sealed with Vaseline.

For the observation of bacterial chain formation on HEp-2 cells, HEp-2 cells were seeded onto a coverslip in a Petri dish and incubated overnight at 37 °C in 5% CO₂. The coverslip with HEp-2 cells was washed with FBS-free medium and incubated for 1 h at 37 °C in 5% CO₂. Subsequently, a bacterial suspension in LB medium was added and incubated for 1 h at 37 °C in 5% CO₂. The coverslip with HEp-2 cells was then washed twice with FBS-free medium and assembled into a tunnel chamber using double-sided tape (~90 µm thick; NW-5, Nichiban, Japan). The ends of the chamber were sealed with Vaseline.

### Flow experiment

The flow chamber was assembled by taping a coverslip to a glass slide, as described previously^25^. The glass slide was bored using a high-speed drill press equipped with a diamond-tipped bit (1 mm diameter, No. 13853; NAKANISHI, Japan) for the inlet and outlet ports. The central portion of the double-sided tape (7082; Teraoka, Japan) was cut to a length of 25 mm long and 1.5 mm wide. The chamber was assembled by taping a coverslip onto a glass slide. The inlet and outlet ports (N-333; IDEX Health & Science, USA) were attached to a high-temperature elastic adhesive (Super X No. 8008 Clear, CEMEDINE, Japan). The finished channel of the sample chamber was straight with a width: 1.5 mm, height: 0.1 mm, and length: 25 mm for bright-field microscopy. A syringe pump (Legato 200; Kd Scientific, USA) was connected to the inlet of the flow chamber via a connector and tubing (F-333NX and 1512L; IDEX Health & Science, USA), without branching. Flow was driven by the syringe pump, pushing the medium through the chamber.

Bacterial suspensions in the LB medium were injected into the chamber and incubated for 15 min at room temperature (RT, 24–26 °C). The unattached cells were slowly washed away by LB medium with or without 0.5% methylcellulose (M0512, viscosity: 4000 cP at 2%, Sigma-Aldrich, USA), at a flow rate of 0.1 µL/s for 1 h. The behavior of attached cells under fluid flow was observed at RT. The flow rate of the syringe pump was calibrated to the velocity of the fluid flow near the glass surface at RT. The near-surface flow speed was determined from dispersed cells that moved passively in the flow direction following occasional breakage of multicellular chains at the glass surface. The shear rate at the chamber surface was estimated using the equation described previously^36,37^.

1 $$\dot \gamma \approx \frac{{6Q}{\mu }}{{{wh}}^{2}}\,$$

Where *γ̇* is the shear rate; *Q* is the flow rate; *w* and *h* are the width and height of the flow chamber, respectively. The shear stress is estimated from shear rate and viscosity of the solution,

2 $${\sigma }_{s}\approx \dot \gamma \mu$$

where *σ*_s_ is the shear stress, μ is the viscosity of the solution, assumed to be 1 mPa·s for LB medium without MC and 14 mPa·s for LB medium containing 0.5% MC. The viscosity of LB medium containing 0.5% MC was estimated from the concentration–viscosity correlation shown in the product datasheet for M0512. Methylcellulose solutions are known to exhibit shear-thinning behavior; however, this value falls within the range (~7–20 mPa·s) expected under the shear rates considered here (~10¹–10⁴ s−¹), based on previous rheological studies^38^. Therefore, a constant viscosity of 14 mPa·s was used as a representative value to estimate shear stress.

### Transmission electron microscope

Bacterial cells were washed three times with PBS and fixed with 2% paraformaldehyde and 2.5% glutaraldehyde in 0.1 M phosphate-buffer (pH 7.4) for 24 h at 4 °C. The samples were then post-fixed in osmium fixation solution [1% (w/v) osmium tetroxide, 1.25% (w/v) potassium ferrocyanide, and 5 mM calcium chloride and embedded in 2% (w/v) low-melting-point agarose. After staining the blocks with 0.5% aqueous uranyl acetate, the specimens were dehydrated and embedded in Spurr resin. Thin sections were mounted on copper grids and post-stained with saturated uranyl acetate and lead citrate. The specimens were observed using an HT7700 transmission electron microscope (Hitachi High Technologies, Japan).

### Complete genome sequencing

Genomic DNA was extracted using the KingFisher Duo Prime System (Thermo Fisher Scientific, USA). For short-read sequencing, the extracted DNA was prepared libraries using the QIAseq FX DNA Library Kit (Qiagen, Germany) and paired-end (150 × 2) sequencing was performed on the Illumina platform. For long-read sequencing, libraries prepared using a Rapid Barcoding Kit V14 (Oxford Nanopore Technologies, UK) were sequenced on the MiniON Mk1B device. Sequencing reads were assembled using Hybracter v0.11.0^39^. Among the plasmid contigs obtained after assembly, only the plasmid containing the *eibG*-like gene was selected for inclusion in the final complete genome sequences. The quality of the assembled genomes was assessed using CheckM2 v1.1.0^40^. Gene annotation was performed using the DFAST platform provided by DNA Data Bank of Japan (https://dfast.ddbj.nig.ac.jp)^41^.

### Phylogenetic analysis of EibG-like proteins

EibG-like proteins were searched in the in-house clinical isolates genome database (not publicly available) using BLAST+ v2.16.0^42^. Based on these results, 13 representative strains harboring EibG-like protein candidates were selected and subjected to complete genome sequencing. EibG-like proteins were re-identified by BLASTp searches using the complete genome sequences of the 13 strains in the database. After removing signal peptides using SignalP v6.0^43^ (https://services.healthtech.dtu.dk/services/SignalP-6.0/), the identified EibG-like proteins, Saa, and YadA were aligned using MAFFT v7.525^44^, processed with trimAl v1.5^45^, and subsequently used to construct a phylogenetic tree with IQ-TREE v3.0.1^46^.

### Visualization of genomic context

To visualize the genomic context, the target gene sequences were obtained, including those 10 kbp upstream and downstream. The gene cluster comparison figure was generated using clinker and clustermap.js v0.0.31^47^.

### In silico structure prediction

The three-dimensional structure of the EibG-like proteins was predicted using AlphaFold3 (DeepMind) via the AlphaFold Server (https://alphafoldserver.com)^48^. The amino acid sequence without the signal peptide removed by SignalP 6.0 was used as the input. The predicted structures were visualized using UCSF ChimeraX v1.10.1^49^.

### Immunoblotting

The culture medium was mixed with Sample Buffer Solution with 3-Mercapto-1,2-propanediol (×4) (FUJIFILM Wako Pure Chemical, Japan) and heated at 50 °C for 10 min. The protein solution was electrophoresed at 20 mA for 75 min on a SuperSep Ace, 5–20% gel (FUJIFILM Wako Pure Chemical, Japan), then transferred to a PVDF membrane at 200 mA for 60 min. The membrane was blocked with EveryBlot Blocking Buffer (Bio-Rad, USA) for 5 min, then reacted with 20 ng/mL Peroxidase ChromPure Human IgG, Fc fragment (Jackson ImmunoResearch, USA).

### Complement sensitivity assay

Overnight cultures in LB medium supplemented with ampicillin were harvested and washed with PBS. MG1655 harboring either an empty vector (negative control) or plasmids expressing *eib* or *cla* (test groups) were resuspended in PBS at a concentration of 5 × 10^8^ CFU/mL. The bacterial suspensions were then incubated with 6.25% non-heated human complement serum (S1764, Sigma-Aldrich, USA) at 37 °C for 60 min with shaking. To confirm complement-dependent killing, heat-inactivated serum (56 °C for 30 min) was used in preliminary experiments to ensure the survival of the negative control strain. Bacterial survival was evaluated by spotting 3 μL of tenfold serial dilutions of the reaction mixture onto LB agar plates containing ampicillin.

### Animal experiment

Animal experiments were performed in the biosafety level 2 animal facility, in accordance with the guidelines established by the Animal Care and Use Committee of The Jikei University School of Medicine (approval no. 2025-005). Three-week-old female SPF BALB/cAJc1 mice were purchased (CLEA Japan, Japan). To avoid the potential influence of sex differences, only female mice were used. A total of 56 mice were used for the infection studies. The animals were randomly divided into two experimental groups (*n* = 31 for O91 Δ*stx1* and *n *= 25 for O91Δ*stx1*Δ*eibG*) and housed in cages (5–6 animals per cage). They were provided with sterilized food, bedding, and water ad libitum throughout the experimental period. The animals were maintained under controlled environmental conditions with a temperature of 22 ± 2 °C, a relative humidity of 55 ± 10%, and a 12:12-h light/dark cycle (lights on from 07:00 to 19:00). Mice were challenged by i.p. injection with a 150 μL bacterial suspension containing approximately 1.5 × 10^8^ colony-forming-units of either O91 Δ*stx1* or O91Δ*stx1*Δ*eibG*. Survival was monitored and recorded every 24 h for 5 days. The infection experiments were replicated at least three times independently.

### Ethical approval

The animal experiments were carried out according to a protocol approved by the Institutional Animal Care and Use Committee of the Jikei University (approval no. 2025-005).

### Statistics and reproducibility

All biological experiments were independently repeated at least three times with similar results. Representative micrographs were chosen from independent experiments that yielded consistent observations.

### Reporting summary

Further information on research design is available in the Nature Portfolio Reporting Summary linked to this article.

## Supplementary information


Supplementary Information
Description of Additional Supplementary Files
Supplementary Data 1 - 3
Movie S1
Movie S2
Movie S3
Movie S5
Movie S4
Movie S6
Movie S7
Movie S8
Reporting Summary
Transparent Peer Review file


## Source data


Source Data


## Data Availability

The sequencing data generated in this study have been deposited in the DNA Data Bank of Japan under accession number PRJDB37405. Source Data are provided with this paper. The published accession numbers used in this study are as follows: ADJ17706.1 (EibG) [https://www.ncbi.nlm.nih.gov/protein/ADJ17706.1/], WP_032488477.1 (YadA) [https://www.ncbi.nlm.nih.gov/protein/WP_032488477.1/], AAL26283.1 (Saa) [https://www.ncbi.nlm.nih.gov/protein/AAL26283.1], PRJNA315192 (routine surveillance at UKHSA) [https://www.ncbi.nlm.nih.gov/bioproject/PRJNA315192/] Source data are provided with this paper.
