# Supplementary material for: Trimeric autotransporter adhesins driving chain-like adhesion diversify surface colonization strategies in Shiga toxin-producing Escherichia coli
Source: Nat Commun. 2026 Aug 4;17:9350. doi: 10.1038/s41467-026-76304-x (PMC13534694; doi:10.1038/s41467-026-76304-x)
Supplement: Supplementary file 1 [file 41467_2026_76304_MOESM12_ESM.pdf]

nature portfolio

Corresponding author(s): Yuto Kotaka

Last updated by author(s): Jun 23, 2026

Reporting Summary

Nature Portfolio wishes to improve the reproducibility of the work that we publish. This form provides structure for consistency and transparency in reporting. For further information on Nature Portfolio policies, see our Editorial Policies and the Editorial Policy Checklist.

Statistics

For all statistical analyses, confirm that the following items are present in the figure legend, table legend, main text, or Methods section.

n/a	Confirmed	
☐	☒	The exact sample size (*n*) for each experimental group/condition, given as a discrete number and unit of measurement
☐	☒	A statement on whether measurements were taken from distinct samples or whether the same sample was measured repeatedly
☐	☒	The statistical test(s) used AND whether they are one- or two-sided<br>*Only common tests should be described solely by name; describe more complex techniques in the Methods section.*
☒	☐	A description of all covariates tested
☐	☒	A description of any assumptions or corrections, such as tests of normality and adjustment for multiple comparisons
☐	☒	A full description of the statistical parameters including central tendency (e.g. means) or other basic estimates (e.g. regression coefficient) AND variation (e.g. standard deviation) or associated estimates of uncertainty (e.g. confidence intervals)
☐	☒	For null hypothesis testing, the test statistic (e.g. *F*, *t*, *r*) with confidence intervals, effect sizes, degrees of freedom and *P* value noted<br>*Give P values as exact values whenever suitable.*
☒	☐	For Bayesian analysis, information on the choice of priors and Markov chain Monte Carlo settings
☒	☐	For hierarchical and complex designs, identification of the appropriate level for tests and full reporting of outcomes
☒	☐	Estimates of effect sizes (e.g. Cohen's *d*, Pearson's *r*), indicating how they were calculated

Our web collection on statistics for biologists contains articles on many of the points above.

Software and code

Policy information about availability of computer code

Data collection	ICCapture, MinKNOW v24.11.8.
Data analysis	Fiji software (ImageJ 1.53t), MinKNOW v24.11.8, Hybracter v0.11.0, CheckM2 v1.1.0, DFAST online, BLAST+ v2.16.0, SignalP v6.0, MAFFT v7.525, trimAl v1.5, IQ-TREE v3.0.1, clinker and clustermap.js v0.0.31, AlphaFold3 server, UCSF ChimeraX v1.10.1, R4.3.2, Microsoft Excel for Mac<br><br>No custom code or software was used for data collection and analysis.

For manuscripts utilizing custom algorithms or software that are central to the research but not yet described in published literature, software must be made available to editors and reviewers. We strongly encourage code deposition in a community repository (e.g. GitHub). See the Nature Portfolio guidelines for submitting code & software for further information.

Data

Policy information about availability of data

All manuscripts must include a data availability statement. This statement should provide the following information, where applicable:

- Accession codes, unique identifiers, or web links for publicly available datasets
- A description of any restrictions on data availability
- For clinical datasets or third party data, please ensure that the statement adheres to our policy

The sequencing data presented in in this study have been deposited in in the DNA Data Bank of of Japan, accession number PRJDB37405.

Research involving human participants, their data, or biological material

Policy information about studies with human participants or human data. See also policy information about sex, gender (identity/presentation), and sexual orientation and race, ethnicity and racism.

Reporting on sex and gender	n/a
Reporting on race, ethnicity, or other socially relevant groupings	n/a
Population characteristics	n/a
Recruitment	n/a
Ethics oversight	The bacterial strains used in this study were clinical isolates provided by the EHEC Working Group, Japan. All samples were anonymized.

Note that full information on the approval of the study protocol must also be provided in the manuscript.

Field-specific reporting

Please select the one below that is the best fit for your research. If you are not sure, read the appropriate sections before making your selection.

☒ Life sciences ☐ Behavioural & social sciences ☐ Ecological, evolutionary & environmental sciences

For a reference copy of the document with all sections, see nature.com/documents/nr-reporting-summary-flat.pdf

Life sciences study design

All studies must disclose on these points even when the disclosure is negative.

Sample size	No statistical methods were used to pre-determine sample size. Sample sizes for animal experiments and microscopy observations were determined based on previous literature and experience in the field to ensure reproducibility.
Data exclusions	One experimental group was excluded from the final analysis because the post-infection observation time points were inconsistent with the protocol applied to all other samples, preventing a direct comparison.
Replication	The findings were reproducible across independent experiments. Each in vitro experiment (including time-lapse microscopy, flow assays, and infection assays) was repeated at least three times using independent biological replicates, yielding consistent results. For animal experiments, the observed phenotypes were confirmed in multiple independent cohorts, with consistent trends across valid replicates.
Randomization	For animal experiments, mice were age-matched and randomly assigned to experimental and control groups. For the HEp-2 cell infection assays and flow experiments, samples were processed in parallel, and fields of view for microscopy were selected randomly to ensure unbiased data collection. No specific randomization method was applied to the genomic sequence analysis as all available sequences meeting the inclusion criteria were included.
Blinding	Blinding was not performed during data collection; however, the potential for observer bias was minimized by using automated image analysis software (Fiji/ImageJ). All quantitative measurements were performed using predefined algorithms that were applied consistently across all experimental groups.

Reporting for specific materials, systems and methods

We require information from authors about some types of materials, experimental systems and methods used in many studies. Here, indicate whether each material, system or method listed is relevant to your study. If you are not sure if a list item applies to your research, read the appropriate section before selecting a response.

Materials & experimental systems

n/a	Involved in the study
	[x] Antibodies
	[x] Eukaryotic cell lines
[x]	Palaeontology and archaeology
	[x] Animals and other organisms
[x]	Clinical data
[x]	Dual use research of concern
[x]	Plants

Methods

n/a	Involved in the study
[x]	ChIP-seq
[x]	Flow cytometry
[x]	MRI-based neuroimaging

Antibodies

Antibodies used	ChromPure Human IgG, Fc Fc fragment (AB_2337046; 009-000-008) was purchased by by Jackson ImmunoResearch Laboratories, Inc.
Validation	The binding specificity of the human IgG Fc fragment was validated in this study by Western blotting (ligand-binding assay).Wild-type STEC strains were used as positive controls, while eibG-deleted mutants (!eibG) served as negative controls. The complete loss of signal in the deletion strain confirms that the interaction is specific to EibG in the context of E. coli whole-cell lysates.

Eukaryotic cell lines

Policy information about cell lines and Sex and Gender in Research

Cell line source(s)	HEp-2 cells: Obtained from an established laboratory legacy collection (original source unknown).
Authentication	The identity of the cell line was verified by its characteristic morphology and its specific adherence pattern (CLAP) in response to STEC infection, consistent with established HEp-2/HeLa characteristics.
Mycoplasma contamination	The cell line was not tested for mycoplasma contamination.
Commonly misidentified lines (See ICLAC register)	HEp-2 is a known HeLa-contaminant. It was used in this study as a standardized host cell model for STEC adherence assays.

Animals and other research organisms

Policy information about studies involving animals; ARRIVE guidelines recommended for reporting animal research, and Sex and Gender in Research

Laboratory animals	Three-week-old female specific pathogen-free (SPF) BALB/c mice (CLEA Japan, Tokyo, Japan).
Wild animals	The study did not involve wild animals.
Reporting on sex	Female mice were intentionally selected for model stability and to reduce inter-animal aggression. Sex was confirmed by the supplier (CLEA Japan) prior to purchase. Sex-based comparative analysis was not performed.
Field-collected samples	The study did not involve samples collected from the field.
Ethics oversight	The animal experiments were carried out according to a protocol approved by the Institutional Animal Care and Use Committee of The Jikei University.

Note that full information on the approval of the study protocol must also be provided in the manuscript.

Plants

Seed stocks	n/a
Novel plant genotypes	n/a
Authentication	n/a
