# Supplementary material for: Trimeric autotransporter adhesins driving chain-like adhesion diversify surface colonization strategies in Shiga toxin-producing Escherichia coli
Source: Nat Commun. 2026 Aug 4;17:9350. doi: 10.1038/s41467-026-76304-x (PMC13534694; doi:10.1038/s41467-026-76304-x)
Supplement: Supplementary file 2 [file 41467_2026_76304_MOESM13_ESM.pdf]

nature portfolio

Peer Review File

Trimeric autotransporter adhesins driving chain-like adhesion diversify surface colonization strategies in Shiga toxin-producing *Escherichia coli*

Corresponding Author: Mr Yuto Kotaka

This file contains all reviewer reports in order by version, followed by all author rebuttals in order by version.

Version 0:

Reviewer comments:

Reviewer #1

(Remarks to the Author)
Kotaka et al. present a manuscript examining the chain-like adhesion phenotype (CLAP) mediated by the trimeric autotransporter adhesin EibG. CLAP has been demonstrated for this protein before, but its relevance to pathogenesis has not been investigated. In the manuscript, the authors demonstrate convincingly that chains adhere more strongly under flow conditions, and that at high flow/shear rates, chains tend to break leaving part of the chain attached while part is swept away, possibly as a mechanism for dispersal. In addition, the authors demonstrate that CLAP and immunoglobulin binding are mediated by different parts of the protein (though this is unsurprising, as the adhesion/chain formation and Ig binding activities were previously shown to be in distinct regions of a related protein, EibD). They also demonstrate the EibG is a virulence factor of STEC using a a mouse infection model, something which has been suspected but not proven before. The authors further investigated the distribution of EibG and related proteins in STEC strains and were able to detect novel CLAP-mediating proteins that are not part of the Eib family.

The study is well designed and presented, though I do have some comments below. My main cricism regarding the study is the claim of CLAP enhancing pathogenesis. This may well be, but what is not demonstrated here is a clear advantage of CLAP over clump formation, which is a much more typical TAA activity. To do this, the authors should perform their flow experiments with strains expressing proteins known to mediate clump formation, such as YadA or EibA, and extract data regarding the relative efficiency of adhesion and partial release of chains vs. clumps under the same flow conditions.

In addition, I have the following comments:

1. Please use the term trimeric autotransporter adhesin (TAA) or type 5c secretion systems, rather than the obsolete OCA.
2. Lines 29 & 32: gene names in italics.
3. There are several small grammar errors here and there. Examples include line 48 (virulence factors), 52 (serogroups, a similar), line 160 (autoaggregation). Please check the manuscript carefully to correct these.
4. Line 55: the adherence pattern in CLAP is not really similar to classical EAEC, which forms a stacked brick-like adhesion pattern.
5. Line 68: it should be mentioned that the Ig binding activity of other Eibs has been mapped to the coiled coil stalk (this is discussed elsewhere, but should be added here).
6. Line 74: TAA-mediated chain formation (as not only EibG does this).
7. Flow experiments: as I understand it, only adhesion to a glass surface was tested. In previous studies, it was shown that Eibs mediate strong attachment to polystyrene surfaces. I do not expect the authors to perform additional experiments regarding this, but it could be raised in the discussion.
8. Line 163: The authors did not detect Ig-binding activity in cell lysates of Cla-expressing cells. However, they note that they boiled the bacteria. Though TAAs tend to be stable, it could be that the Cla trimers are disrupted at this temperature and any Ig-binding activity is lost. I therefore recommend only heating samples to 50 degrees to prevent disassociation of trimers. It might also be prudent to perform this experiment using isolated outer membranes rather than whole-cell lysates. Please include an image of the corresponding SDS-PAGE gel to demonstrate equal loading and expression of the TAAs.
9. Lines 187-192: There is some confusion here, as the study examining IgG binding was performed on EibD, not EibE.
Lines 195 & 262: the Δsaddle mutant lacks the entire passenger (up to and including the saddle); the way it is phrased here

is misleading.
Line 339: This section may need clarification. Surely the bacteria were diluted in MEM before adding to the HEP-2 cells? What was the M.O.I. in these experiments?
Line 381: The authors mention a plasmid with an eibG-like sequence. This could be expanded on in the main text, as other eib genes are found in mobile genetic elements.
Figure 4C: The structure suggested to be YadA is not the Yersinia YadA structure. Please correct or clarify.
Figure 6: What does the shading around the curves indicates? Confidence intervals? Please clarify.

Reviewer #2

(Remarks to the Author)
The study by Kotaka et al investigated the dynamics of chain like adherence pattern (CLAP) in STEC mediated by the trimeric autotransporter adhesin (TAA) EibG.
Many TAA proteins are involved in biofilm formation, auto-aggregation, host cell adherence and virulence (Linke et al, 2006. DOI: 10.1016/j.tim.2006.04.005) (Kiessling 2019. DOI DOI: https://doi.org/10.1007/s00430-019-00652-3). The EIB proteins have multiple homologues and have been previously characterised e.g. (Leo et al, 2011. DOI: 10.1016/j.str.2011.03.021), (Swiatek et al, 2025. DOI: https://doi.org/10.1038/s41598-025-22967-3), (Leo et al 2009. DOI: 10.1016/j.molimm.2009.02.024), (Merkel et al, 2010. DOI: https://doi.org/10.1128/iai.00006-10), (Sandt et al, 2000. DOI https://doi.org/10.1128/iai.68.4.2205-2214.2000)
The authors show that the CLAP phenotype originates from single cells that elongate and divide without separating. These chains can break apart under flow to release viable cells that then disperse. The authors also identified more EibG like proteins using genomics and show that these EibG like proteins can mediate CLAP but have predicted structural differences and do not bind to IgG. Finally, the authors demonstrate that mice infected with STEC lacking eibG have increased survival compared to mice infected with the parent strain. The major strength of this paper are the results in figure 3 and the paper would be strengthened by further exploration or focus on these observations. However, the animal model used in figure 6 is inappropriate for assessing the role of an adhesin to virulence and there are some questions regarding the significance/novelty of the results in figures 4 and 5 without further experiments.

Major comments
1. ClaA and ClaB did not show any IgG binding in the Western blot. However, there were no loading controls for this blot and so expression levels may be different between samples. An ELISA could be used as an alternative method that is more sensitive than Western blot.
2. There is a pairwise analysis of the overall amino acid sequence similarity comparing EibG and identified homologues but given the lack of IgG binding of ClaA and B, it would be nice to see if there are obvious mutations at the predicted IgG binding sites in ClaA/B to understand this further. Could this be validated by introducing mutations into EibG and show this results in loss of IgG binding?
3. Do the ClaA and B proteins confer resistance to complement killing?
4. More information could be given about the 41 eibG homologues that were identified. If these were identified from 14 genomes, that must mean some strains had multiple eibG like proteins? Were these chromosomal or plasmid encoded? Can there be anything inferred about the evolution of eibG-like proteins?
5. The animal model used is not the most appropriate for assessing the function of an adhesin in vivo. This model only has a death readout. Ideally, an oro-gastric route of infection could have been used and then you can assess the role of EibG in attaching to the host gastrointestinal tract and whether this influences infection. You could also assess bacterial burdens along the gastrointestinal tract. This would be a more direct assay for understanding the role of EibG in vivo.

Minor comments
1. Some of the figures are quite small with small fonts (Fig 3A, C E and G. Fig. 4A and B). Increased font size and/or figure size would help with this.
2. The manuscript does not refer to trimeric autotransporter adhesins (TAA) or state that EibG is a TAA/member of the type 5 secretion system. This does not necessarily detract from the data, but it would add context and bring about a broader audience who have interest in type 5 secreted proteins or TAA's.
3. There is a discrepancy with what is labelled as ClaB in the neighbourhood analysis and phylogenetic tree. It would also be worthwhile showing which sequence was used to generate the Alphafold models in Figure 4c.
4. The predicted structure of YadA looks quite different to what has been previously published (Swiatek et al, 2025, DOI https://doi.org/10.1038/s41598-025-22967-3) especially the head domain. Does this potentially affect the predicted structures for the other proteins shown in figure 4?

Reviewer #3

(Remarks to the Author)

The study by Yuto Kotaka and collaborators presents several noteworthy findings, most prominently the identification of the molecular origin of the segmented filaments observed with STEC E. coli cells, specifically pinpointing the adhesin responsible for this distinctive phenotype. Additionally, the work places this class of adhesins within a broader phylogenetic context, offering insights into their evolutionary history. Functionnal significance is briefly discussed, with references to

similar phenotypes observed in other pathogenic bacteria.

While the study's potential impact on the field and related disciplines appears promising, a comprehensive assessment of its originality and how it compares to the established literature is beyond my domain of expertise.

The conclusions and claims drawn from the microscopy experiments are well-supported by the data presented. However, some aspects of the data analysis and interpretation could benefit from additional clarification to ensure the experimental instructions are fully understandable and reproducible.

More specifically:

- Material listed for microscopy experiments (dark-field, bright-field and fluidic) is coherent with the experiment performed.

- For the Time-lapse imaging of chain formation section of the material and methods
. It is unclear how cells attach to the glass coverslip from the description provided lines 332-336.

- For the Flow experiment section
. Indicate whether the syringe pump is branched so as to pump fluid in or out of the device (positive of negative pressure applied in the chamber)

. It seems relevant to further clarify that the observation of filaments breaking under flow was carried out at RT (what would be RT?)
. This choice can be justified (contribution of cell division machinery quite unlikely in the time window of the experiment, for example?) Clearly stating the advantage and limitations of this unsual temparature setting may become highly relevant in the field.

- data analysis:
. Expand the description of the analysis pipeline presenetd briefly on lines 324-325.
. plug-in name? input parameter values?

- Optical microscopy section
. l.323 (and similarly l.328) why stating 'Projections of the images'? Are images the result of a combination of information from multiple images, like maximal intensity projection?
. Could you confirm, for example, that images presenetd in Figure 2A are single images taken with the x40 objective and dark-field condenser and where simply adjusted to maximize contrasts?

- Shear stress and flow speed measurement:
. Shear stress is not calibrated (Fig S1). The perfect lines are the result of applying a formula, and this is fine, but this is not a calibration (The use of the term calibration is misleading).
. In addition, could you further contextualize the value of 14 mPa.sec used here.
. The relationship between the vendor specification (4000cP at 2% 25°C) and the 14cP mentionned here (0.5%, RT) is unclear.
. You could also add that within the shear rate range considered, methylcellulose solutions should not be considered as a shear-thinning fluids to avoid confusion with widely difuused information about MC when used for other purposes.
In fact, a clarification of the relationship between flow rate, shear rate and shear stress may be welcome and could be associated with Figure S1.

other elements:
- gene names to be italicized (even in the abstract)
- l.106 'Figure 3B, C' instead of 'Figure 3BC'
- l.114 shear stress rather than FLOW rate
- sup Fig S2: gene name nomenclature dictates that the names on the left side of the heatmap should be italicized with a lower case first letter
Or change the legend for virulence factor proteins and probably % similarity instead of identity?
- l.324 "under at 10-s resolution" consider removing 'under'

Version 1:

Reviewer comments:

Reviewer #1

(Remarks to the Author)
The authors have addressed my comments satisfactorily. Importantly, they have performed experiments according my major comment regarding the advantage of CLAP over clump formation and show increased dispersal from chains over clumps - an important finding that is now substantiated.

I still have a couple of minor comments that the authors may wish to address before publication:
- Lines 202-210: A reference should be added for clump formation by EibA (10.1002/mbo3.1109 or 10.1038/s41598-025-22967-3)
- Line 211: Enterococcus should be in italics
- Where was the template for eibA amplification obtained?

Reviewer #2

(Remarks to the Author)
I commend the authors for addressing previously raised concerns, these changes have improved the manuscript and strengthened the authors conclusions. I have no further comments.

Open Access This Peer Review File is licensed under a Creative Commons Attribution 4.0 International License, which permits use, sharing, adaptation, distribution and reproduction in any medium or format, as long as you give appropriate credit to the original author(s) and the source, provide a link to the Creative Commons license, and indicate if changes were made.
In cases where reviewers are anonymous, credit should be given to 'Anonymous Referee' and the source.
The images or other third party material in this Peer Review File are included in the article's Creative Commons license, unless indicated otherwise in a credit line to the material. If material is not included in the article's Creative Commons license and your intended use is not permitted by statutory regulation or exceeds the permitted use, you will need to obtain permission directly from the copyright holder.
To view a copy of this license, visit https://creativecommons.org/licenses/by/4.0/

Point-by-point response to the reviewers' comments

manuscript ID: NCOMMS-25-104706

Reviewer #1 (Remarks to the Author):

Kotaka et al. present a manuscript examining the chain-like adhesion phenotype (CLAP) mediated by the trimeric autotransporter adhesin EibG. CLAP has been demonstrated for this protein before, but its relevance to pathogenesis has not been investigated. In the manuscript, the authors demonstrate convincingly that chains adhere more strongly under flow conditions, and that at high flow/shear rates, chains tend to break leaving part of the chain attached while part is swept away, possibly as a mechanism for dispersal. In addition, the authors demonstrate that CLAP and immunoglobulin binding are mediated by different parts of the protein (though this is unsurprising, as the adhesion/chain formation and Ig binding activities were previously shown to be in distinct regions of a related protein, EibD). They also demonstrate the EibG is a virulence factor of STEC using a a mouse infection model, something which has been suspected but not proven before. The authors further investigated the distribution of EibG and related proteins in STEC strains and were able to detect novel CLAP-mediating proteins that are not part of the Eib family.
The study is well designed and presented, though I do have some comments below. My main cricism regarding the study is the claim of CLAP enhancing pathogenesis. This may well be, but what is not demonstrated here is a clear advantage of CLAP over clump formation, which is a much more typical TAA activity. To do this, the authors should perform their flow experiments with strains expressing proteins known to mediate clump formation, such as YadA or EibA, and extract data regarding the relative efficiency of adhesion and partial release of chains vs. clumps under the same flow conditions.

Response: We appreciate this insightful suggestion. We agree that demonstrating the specific advantages of chain formation over typical clump formation is essential for establishing its biological significance. In response to this comment, we performed additional flow experiments using cells expressing EibA, a well-characterized TAA known to mediate clump formation. As shown in Figure 3G and Movie S8, EibA-mediated clumps exhibited significantly higher adherence than single adherent cells. However, we observed a fundamental mechanistic distinction: the partial release and subsequent dispersal of cells were notably absent in clump-forming strains. These results confirm that this dispersal mechanism is a unique functional attribute specifically enabled by the multicellular chain architecture. We believe that these comparative data provide a clear functional justification for the adaptive significance of CLAP and significantly strengthen the manuscript's overall

argument. We have incorporated these findings into the Results section (Revised lines 146-154).

In addition, I have the following comments:

1. Please use the term trimeric autotransporter adhesin (TAA) or type 5c secretion systems, rather than the obsolete OCA.

Response: We thank the reviewer for this helpful correction. We agree that trimeric autotransporter adhesin (TAA) is the more standard and widely recognized term in the field. While we initially used OCA as a broader descriptor, we have now updated the terminology to TAA throughout the revised manuscript. Please refer to the track changes version of the manuscript to see all instances of this update.

2. Lines 29 & 32: gene names in italics.

Response: Thank you. We have corrected the gene names to be in italics (Revised line 34).

3. There are several small grammar errors here and there. Examples include line 48 (virulence factors), 52 (serogroups, a similar), line 160 (autoaggregation). Please check the manuscript carefully to correct these.

Response: Thank you for your comments. We have corrected the grammar errors throughout the manuscript. Please refer to the track changes version of the manuscript to see all instances of this update.

4. Line 55: the adherence pattern in CLAP is not really similar to classical EAEC, which forms a stacked brick-like adhesion pattern.

Response: Thank you for your comments. We agree with the reviewer that the morphological characteristics of CLAP are distinct from the "stacked brick-like" pattern of EAEC. To ensure a more precise description of the EibG-mediated phenotype and avoid potential confusion, we have removed the description regarding its morphological similarities to EAEC from the Introduction (Revised line 58).

5. Line 68: it should be mentioned that the Ig binding activity of other Eibs has been mapped to the coiled coil stalk (this is discussed elsewhere, but should be added here).

Response: Thank you for pointing that out. We have added a sentence clarifying that the immunoglobulin-binding activity of other members of the Eib family, such as EibD, has been mapped to the coiled-coil stalk region. (Revised lines 64-65).

6. Line 74: TAA-mediated chain formation (as not only EibG does this).

Response: Thank you. As suggested, we have updated the text to TAA-mediated chain formation (Revised line 73).

7. Flow experiments: as I understand it, only adhesion to a glass surface was tested. In previous studies, it was shown that Eibs mediate strong attachment to polystyrene surfaces. I do not expect the authors to perform additional experiments regarding this, but it could be raised in the discussion.

Response: Thank you for your insightful comments. Based on your comments, I have added the discussion to the discussion section (line 321-325).

8. Line 163: The authors did not detect Ig-binding activity in cell lysates of Cla-expressing cells. However, they note that they boiled the bacteria. Though TAAs tend to be stable, it could be that the Cla trimers are disrupted at this temperature and any Ig-binding activity is lost. I therefore recommend only heating samples to 50 degrees to prevent disassociation of trimers. It might also be prudent to perform this experiment using isolated outer membranes rather than whole-cell lysates. Please include an image of the corresponding SDS-PAGE gel to demonstrate equal loading and expression of the TAAs.

Response: We appreciate this constructive technical suggestion. Following the reviewer's recommendation, we have repeated all immunoblotting experiments using a 50°C heating condition instead of boiling to preserve the trimeric state and we have included the corresponding SDS-PAGE gels (whole protein staining) to demonstrate equal loading and high-level expression of all TAAs (EibG, ClaA, and ClaB). Our results show that while clear, high-molecular-weight bands were detected for all TAAs, IgG-Fc binding was exclusively observed for EibG and was consistently absent for ClaA and ClaB. These findings confirm that the lack of IgG-binding activity in Cla proteins is a genuine functional distinction rather than an artifact of sample preparation. Accordingly, we have updated the Figure 5 and Materials and Methods section (Revised line 557).

9. Lines 187-192: There is some confusion here, as the study examining IgG binding was performed on EibD, not EibE.

Response: Thank you for pointing that out. We have revised the text to state that the IgG binding study was performed on EibD (Revised lines 238-244 and 938).

Lines 195 & 262: the Δsaddle mutant lacks the entire passenger (up to and including the saddle); the way it is phrased here is misleading.

Response: Thank you for the correction. We have corrected the description to clarify that the Δsaddle mutant lacks the entire passenger domain up to and including the saddle (Revised lines 240, 246, and 947).

Line 339: This section may need clarification. Surely the bacteria were diluted in MEM before adding to the HEP-2 cells? What was the M.O.I. in these experiments?

Response: Thank you for pointing that out. We have clarified the detailed procedure for the infection experiment in the revised Materials and Methods section (Revised lines 410–415).

Line 381: The authors mention a plasmid with an eibG-like sequence. This could be expanded on in the main text, as other eib genes are found in mobile genetic elements.

Response: Thank you for your comments. In our dataset, *yalA* was the uniquely plasmid-encoded *eibG*-like gene and is phylogenetically related to the established plasmid-mediated adhesin *saa*. Although functional analysis of *yalA* is outside the scope of this study, its plasmid localization serves as direct evidence that this adhesin family undergoes active horizontal gene transfer, contrasting with the chromosomal integration of other TAA members. We have added a discussion regarding these points to the main text (Revised lines 182-186).

Figure 4C: The structure suggested to be YadA is not the Yersinia YadA structure. Please correct or clarify.

Response: Thank you for pointing that out. Following your comment, we performed a comprehensive re-evaluation and reorganization of all structural prediction results and their corresponding labels throughout the manuscript to ensure absolute accuracy and consistency (Revised Figure 4C).

Figure 6: What does the shading around the curves indicates? Confidence intervals? Please clarify.

Response: Thank you for pointing that out. The shading around the Kaplan-Meier curves represents the 95% confidence intervals. We have clarified this in the figure legend of the revised supplementary information.

Reviewer #2 (Remarks to the Author):

The study by Kotaka et al investigated the dynamics of chain like adherence pattern (CLAP) in STEC mediated by the trimeric autotransporter adhesin (TAA) EibG.
Many TAA proteins are involved in biofilm formation, auto-aggregation, host cell adherence and virulence (Linke et al, 2006. DOI: 10.1016/j.tim.2006.04.005) (Kiessling 2019. DOI DOI: https://doi.org/10.1007/s00430-019-00652-3). The EIB proteins have multiple homologues and have been previously characterised e.g. (Leo et al, 2011. DOI: 10.1016/j.str.2011.03.021), (Swiatek et al, 2025. DOI: https://doi.org/10.1038/s41598-025-22967-3), (Leo et al 2009. DOI: 10.1016/j.molimm.2009.02.024), (Merkel et al, 2010. DOI: https://doi.org/10.1128/iai.00006-10), (Sandt et al, 2000. DOI https://doi.org/10.1128/iai.68.4.2205-2214.2000)
The authors show that the CLAP phenotype originates from single cells that elongate and divide without separating. These chains can break apart under flow to release viable cells that then disperse. The authors also identified more EibG like proteins using genomics and show that these EibG like proteins can mediate CLAP but have predicted structural differences and do not bind to IgG. Finally, the authors demonstrate that mice infected with STEC lacking eibG have increased survival compared to mice infected with the parent strain. The major strength of this paper are the results in figure 3 and the paper would be strengthened by further exploration or focus on these observations. However, the animal model used in figure 6 is inappropriate for assessing the role of an adhesin to virulence and there are some questions regarding the significance/novelty of the results in figures 4 and 5 without further experiments.

Major comments
1. ClaA and ClaB did not show any IgG binding in the Western blot. However, there were no loading controls for this blot and so expression levels may be different between samples. An ELISA could be used as an alternative method that is more sensitive than Western blot.
Response: We appreciate this constructive technical suggestion. Following the reviewer's recommendation, we have repeated all immunoblotting experiments using a 50°C heating condition instead of boiling to preserve the trimeric state and we have included the corresponding SDS-PAGE gels (wholw protein staining) to demonstrate equal loading and high-level expression of all TAAs (EibG, ClaA, and ClaB). Our results show that while clear, high-molecular-weight bands were detected for all TAAs, IgG-Fc binding was exclusively observed for EibG and was consistently absent for ClaA and ClaB. These findings confirm that the lack of IgG-binding activity in Cla proteins is a genuine functional distinction rather

than an artifact of sample preparation. Accordingly, we have updated the Figure 5 and Materials and Methods section (Revised line 557).

2. There is a pairwise analysis of the overall amino acid sequence similarity comparing EibG and identified homologues but given the lack of IgG binding of ClaA and B, it would be nice to see if there are obvious mutations at the predicted IgG binding sites in ClaA/B to understand this further. Could this be validated by introducing mutations into EibG and show this results in loss of IgG binding?

Response: We appreciate this insightful suggestion. In accordance with your feedback, we constructed a novel EibG mutant and evaluated its IgG-binding activity. This mutant was designed by incorporating the predicted IgG-binding domain from ClaA/B into EibG, based on multiple sequence alignment. While SDS-PAGE confirmed that the mutant was expressed at the same molecular weight as the wild-type, however its IgG-binding activity was lost. These results lead us to conclude that Cla lacks IgG-binding activity. We have added a results and discussion regarding these points to the main text (Revised lines 250-255).

3. Do the ClaA and B proteins confer resistance to complement killing?

Response: We appreciate this pivotal suggestion. Based on your suggestion, we have examined the resistance to serum (complement) killing. Our experiments confirmed that both ClaA and ClaB confer resistance to complement killing, like EibG and EibA. Furthermore, we demonstrated that complement resistance is retained even in the $EibG^{D391A}$ mutant, which is deficient in IgG-binding activity. These results indicate that complement resistance in Eib and Cla family proteins is a shared functional trait that operates independently of their ability to bind IgG. While the precise molecular mechanism of this resistance remains to be elucidated in future work, these findings highlight a significant functional relationship and commonality between the Eib and Cla proteins. We have included these data in the revised manuscript (Figure 6, Revised lines 267-274).

4. More information could be given about the 41 eibG homologues that were identified. If these were identified from 14 genomes, that must mean some strains had multiple eibG like proteins? Were these chromosomal or plasmid encoded? Can there be anything inferred about the evolution of eibG-like proteins?

Response: Thank you for pointing that out. Among the 13 genomes analyzed, we identified a total of 41 EibG-like homologues, with the number per strain ranging from 1 to 8 copies. This variation in copy number suggests that gene duplication and subsequent

diversification are ongoing processes within this adhesin family. Regarding their genomic localization, while many of these homologues are encoded on the chromosome, we observed interesting evidence of mobility. Specifically, for the variant YalA, we identified copies on both a plasmid (JNEC-KY178) and a chromosome (JNEC-KY180) in different strains. This dual localization, along with the phylogenetic relationship between YalA and the plasmid-encoded adhesin Saa, strongly implies a dynamic evolutionary history involving horizontal gene transfer and inter-replicon mobilization. We have updated the Results section to include these detailed genomic characteristics (Revised lines 174-176, 182-186).

5. The animal model used is not the most appropriate for assessing the function of an adhesin in vivo. This model only has a death readout. Ideally, an oro-gastric route of infection could have been used and then you can assess the role of EibG in attaching to the host gastrointestinal tract and whether this influences infection. You could also assess bacterial burdens along the gastrointestinal tract. This would be a more direct assay for understanding the role of EibG in vivo.

Response: We are grateful for the reviewer's constructive comment. We agree that for a protein categorized as an adhesin, the oro-gastric route is the standard for evaluating intestinal colonization. However, our use of the intraperitoneal (i.p.) route was a deliberate choice intended to capture a different dimension of EibG's biological relevance—specifically its contribution to systemic infection. We realize that we did not sufficiently justify why this model was selected for evaluating a multifunctional protein like EibG, and we are grateful for the opportunity to clarify this.

To address the reviewer's concern, we have revised the manuscript to more explicitly define the scope of our in vivo experiments and the biological relevance of EibG's multifaceted functions (Revised lines 275-280). While these in vivo findings provide important context, we acknowledge that the main subject of this study is the fundamental biophysical characterization of CLAP and the identification of the novel Cla lineage. To ensure the manuscript remains focused on these core mechanisms, we have moved the i.p. infection data to the Supplementary Information (Figure S5). We believe this restructuring clarifies the study's scope and emphasizes our primary findings on surface colonization strategies.

Below, we provide a detailed explanation of why the i.p. route was selected as the most appropriate model for this study.

<u>(i) Clarification of the study scope</u>

The primary objective of this experiment was to assess whether the multifaceted functions of EibG contribute to pathogenicity in a broad biological context, rather than focusing exclusively on its role as an intestinal adhesin. Therefore, the i.p. route was selected as the most appropriate model to evaluate these integrated functions.

(ii) Limitations of the mouse oral infection model

Although the natural infection route of STEC is oral, our preliminary experiments using an ICR mouse model (10^3 CFU) did not yield results suitable for detailed analysis. While the bacteria were stably recovered from feces, SEM observation of the intestinal lumen failed to detect any clear attachment to the epithelial cells. This outcome is consistent with the established consensus in the field that STEC strains often do not robustly colonize the gastrointestinal tract, in contrast to the well-established *Citrobacter rodentium* model (Mullineaux-Sanders, C et al., *Nat Rev Microbiol*, 2019).

(iii) EibG as a factor in systemic pathogenicity

We noted that Eib protein homologues are occasionally found in extraintestinal pathogenic E. coli isolated from blood or urine (e.g., GenBank: GCA_050828455.1 and GCA_044001315.1), where they have been suspected of playing a role in virulence (Krawczyk B et al., *Int J Mol Sci*, 2025). By employing the i.p. route, we were able to evaluate EibG's pathogenicity in this broader context. As shown in our new data (Figure 6), EibG and Cla confer robust resistance to complement-mediated killing. The i.p. model allowed us to link EibG's multifaceted functions (e.g., adhesion, IgG binding, and serum resistance) to a clear, reproducible, and quantifiable endpoint: lethality. This demonstrates that EibG contributes to bacterial survival and virulence within the host environment, independent of Shiga toxin.

Minor comments

1. Some of the figures are quite small with small fonts (Fig 3A, C E and G. Fig. 4A and B). Increased font size and/or figure size would help with this.

Response: Thank you for this practical suggestion to improve readability. In response to your comment, we have updated Figures 3 and 4 with larger fonts to improve readability. Additionally, we are prepared to provide high-resolution image files to ensure optimal clarity for the publication.

2. The manuscript does not refer to trimeric autotransporter adhesins (TAA) or state that EibG is a TAA/member of the type 5 secretion system. This does not necessarily detract

from the data, but it would add context and bring about a broader audience who have interest in type 5 secreted proteins or TAA's.

Response: We thank the reviewer for this helpful correction. We agree that trimeric autotransporter adhesin (TAA) is the more standard and widely recognized terminology in the field. While we initially used OCA as a broader descriptor, we have now updated the terminology to TAA throughout the revised manuscript. Please refer to the track changes version of the manuscript to see all instances of this update.

3. There is a discrepancy with what is labelled as ClaB in the neighbourhood analysis and phylogenetic tree. It would also be worthwhile showing which sequence was used to generate the Alphafold models in Figure 4c.

Response: Thank you for pointing that out. We have corrected and standardized the labeling of ClaA and ClaB to ensure consistency between the neighborhood analysis and the phylogenetic tree (Revised Figure 4B). For complete transparency and reproducibility, we have included the exact amino acid sequence used for this prediction in Source Data.

4. The predicted structure of YadA looks quite different to what has been previously published (Swiatek et al, 2025, DOI https://doi.org/10.1038/s41598-025-22967-3) especially the head domain. Does this potentially affect the predicted structures for the other proteins shown in figure 4?

Response: Thank you for pointing that out. Following your comment, we performed a comprehensive re-evaluation and reorganization of all structural prediction results and their corresponding labels throughout the manuscript to ensure absolute accuracy and consistency (Revised Figure 4C).

Reviewer #3 (Remarks to the Author):

The study by Yuto Kotaka and collaborators presents several noteworthy findings, most prominently the identification of the molecular origin of the segmented filaments observed with STEC E. coli cells, specifically pinpointing the adhesin responsible for this distinctive phenotype. Additionally, the work places this class of adhesins within a broader phylogenetic context, offering insights into their evolutionary history. Functionnal significance is briefly discussed, with references to similar phenotypes observed in other pathogenic bacteria.

While the study's potential impact on the field and related disciplines appears promising, a comprehensive assessment of its originality and how it compares to the established literature is beyond my domain of expertise.
The conclusions and claims drawn from the microscopy experiments are well-supported by the data presented. However, some aspects of the data analysis and interpretation could benefit from additional clarification to ensure the experimental instructions are fully understandable and reproducible.

More specifically:
- Material listed for microscopy experiments (dark-field, bright-field and fluidic) is coherent with the experiment performed.
Response: Thank you for carefully checking the materials listed for the microscopy experiments.

- For the Time-lapse imaging of chain formation section of the material and methods
. It is unclear how cells attach to the glass coverslip from the description provided lines 332-336.
Response: Thank you for your comment. We have clarified in the revised manuscript that cells were allowed to attach nonspecifically to the untreated glass surface during the incubation period (10 min at 37°C). This description has been added to improve clarity (Revised lines 458–459).

- For the Flow experiment section
. Indicate whether the syringe pump is branched so as to pump fluid in or out of the device (positive of negative pressure applied in the chamber)
Response: Thank you for your comment. We have clarified that the syringe pump was connected to the inlet of the flow chamber without branching and operated in positive pressure mode, pushing the medium through the device. The corresponding description has been added to the revised manuscript (Revised lines 476–478).

. It seems relevant to further clarify that the observation of filaments breaking under flow was carried out at RT (what would be RT?). This choice can be justified (contribution of cell division machinery quite unlikely in the time window of the experiment, for example?) Clearly stating the advantage and limitations of this unusual temparature setting may become highly relevant in the field.

Response: Thank you for your insightful comments. We have clarified that the observations were conducted at room temperature (RT, 24–26 °C), and added this information to the revised manuscript (Revised lines 479–480). While cell division still occurs at RT, the short observation window (~1 min) limits its contribution during chain breakage observations. We have also added a brief statement in the revised manuscript to clarify the advantages and limitations of this temperature condition (Revised lines 128–129).

- data analysis:

. Expand the description of the analysis pipeline presenetd briefly on lines 324-325.

. plug-in name? input parameter values?

Response: Thank you for your comment. Image straightening in Fig. 2A was performed using the built-in Straighten function in Fiji (ImageJ, version 1.53t). For cell area analysis in Fig. 3C, we used the built-in Analyze Particles function in Fiji after thresholding (intensity range: 0–102) without applying size or circularity filters. Chain breakage events in Fig. 3E were manually counted, defined as the separation of multicellular chains consisting of more than three connected cells into two or more fragments. We have added a detailed description of the data analysis pipeline in the revised manuscript (Revised lines 434–436, 439–443).

- Optical microscopy section

. l.323 (and similarly l.328) why stating 'Projections of the images'? Are images the result of a combination of information from multiple images, like maximal intensity projection?

Response: Thank you for your comment. We apologize for the ambiguity caused by the term “projections.” The images were acquired as time-lapse sequences of single-frame images, and no image projection or stacking (e.g., maximal intensity projection) was performed. We have revised the manuscript accordingly (Revised lines 434–436, 439–443).

. Could you confirm, for example, that images presenetd in Figure 2A are single images taken with the x40 objective and dark-field condenser and where simply adjusted to maximize contrasts?

Response: Thank you for your comment. The images presented in Figure 2A are single images acquired using a ×40 objective with a dark-field condenser and are only adjusted to optimize contrast (pseudo colored for visualization). This has been clarified in the revised manuscript (Revised line 871).

- Shear stress and flow speed measurement:

. Shear stress is not calibrated (Fig S1). The perfect lines are the result of applying a formula, and this is fine, but this is not a calibration (The use of the term calibration is misleading).

Response: Thank you for your comment. We agree that the term "calibration" was inappropriate in this context. The shear stress was not experimentally calibrated but estimated based on the analytical expression described previously. We have revised the title of Figure S1 from "Calibration of flow dynamics" to "Flow speed characterization and estimation of shear stress" accordingly (Supplementary Information).

. In addition, could you further contextualize the value of 14 mPa.sec used here.

. The relationship between the vendor specification (4000cP at 2% 25°C) and the 14cP mentionned here (0.5%, RT) is unclear.

Response: Thank you for your comment. The viscosity value (14 mPa·s) was estimated from the concentration–viscosity correlation graph shown in the product datasheet for M0512 (Sigma-Aldrich). The datasheet is available online at https://www.sigmaaldrich.com/deepweb/assets/sigmaaldrich/product/documents/251/785/m0512pis.pdf. This graph reflects the strong, non-linear dependence of viscosity on polymer concentration, which explains the decrease from the vendor specification (4,000 cP at 2%) to the value at 0.5%. We have clarified this in the revised manuscript (Revised lines 495–496).

. You could also add that within the shear rate range considered, methylcellulose solutions should not be considered as a shear-thinning fluids to avoid confusion with widely difuused information about MC when used for other purposes.

Response: Thank you for your comment. We agree that methylcellulose solutions can exhibit shear-thinning behavior. In the present study, the estimated shear rate ranged from ~10^1 to 10^4 s^{-1}, within which non-Newtonian effects may be present, particularly at higher shear rates. However, for the purpose of estimating shear stress, the solution was approximated as a Newtonian fluid within this shear rate range. Therefore, in the context of this study, the solution was not treated as a shear-thinning fluid, and this approximation does not affect the qualitative conclusions of this study. We have added this clarification to the revised manuscript (Revised lines 496–498).

In fact, a clarification of the relationship between flow rate, shear rate and shear stress may be welcome and could be associated with Figure S1.

Response: Thank you for your comment. We have clarified the relationship between flow rate, shear rate, and shear stress in the revised manuscript (Revised line 494-499) and in the legend of Figure S1. The shear rate was estimated as $\dot{\gamma} \approx 6Q/(wh^2)$, and the shear stress was calculated as $\sigma = \mu\dot{\gamma}$.

other elements:

- gene names to be italicized (even in the abstract)

Response: Thank you. We have revised the manuscript accordingly (Revised line 34).

- l.106 'Figure 3B, C' instead of 'Figure 3BC'

Response: Thank you. We have revised the manuscript accordingly (Revised line 121).

- l.114 shear stress rather than FLOW rate

Response: Thank you for your comment. We have revised the sentence in line 131 to "shear stress rather than flow rate."

- sup Fig S2: gene name nomenclature dictates that the names on the left side of the heatmap should be italicized with a lower case first letter
Or change the legend for virulence factor proteins and probably % similarity instead of identity?

Response: Thank you. We have revised the manuscript accordingly (Revised Figure S2).

- l.324 "under at 10-s resolution" consider removing 'under'

Response: Thank you for your comment. We have revised the sentences in lines 432 to "Time-lapse images were acquired every 10 s."

Point-by-point response to the reviewers' comments v2

manuscript ID: NCOMMS-25-104706A

Reviewer #1 (Remarks to the Author):

- Lines 202-210: A reference should be added for clump formation by EibA (10.1002/mbo3.1109 or 10.1038/s41598-025-22967-3)

Response: We agree with the reviewer that adding a reference here strengthens the statement regarding EibA-mediated clump formation. We have added the suggested reference (10.1038/s41598-025-22967-3) to the text.

Location in Manuscript: We revised lines 128-129 as follows.

"To clarify the advantages of CLAP under shear stress, we compared the adherence dynamics of EibG-mediated chains with those of EibA-mediated clumps using our flow assay system [23]."

- Line 211: Enterococcus should be in italics

Response: We apologize for this formatting oversight. *Enterococcus* has now been italicized as requested.

Location in Manuscript: We have changed the font of line 137 to italics.

- Where was the template for *eibA* amplification obtained?

Response: Thank you for pointing out this missing detail in our methodology. The template DNA for *eibA* amplification was obtained from *Escherichia coli* strain ECOR-9. We have clarified this in the Supplementary Table S1.

Location in Manuscript: We have added a "DNA template" column to Supplementary Table 3 and clearly stated that *eibA* was amplified from the ECOR-9 strain.
