# Supplementary material for: Trimeric autotransporter adhesins driving chain-like adhesion diversify surface colonization strategies in Shiga toxin-producing Escherichia coli
Source: Nat Commun. 2026 Aug 4;17:9350. doi: 10.1038/s41467-026-76304-x (PMC13534694; doi:10.1038/s41467-026-76304-x)
Supplement: Supplementary file 3 [file 41467_2026_76304_MOESM1_ESM.pdf]

Supplementary Informations

Trimeric autotransporter adhesins driving chain-like adhesion diversify surface colonization strategies in Shiga toxin-producing *Escherichia coli*

Yuto Kotaka[1, 2 *], Naoki A Uemura[3], Tadayuki Iwase[4, 5], Kenichi Lee[2], Nozomi Ishijima[2], Daisuke Nakane[3], Hirotaka Kobayashi[6], Michiyo Kataoka[6], Yukihiro Akeda[2, 7, 8, 9], Sunao Iyoda[2]

[1] Department of Biological Sciences, Graduate School of Science, Tokyo Metropolitan University, Tokyo, Japan

[2] Department of Bacteriology I, National Institute of Infectious Diseases, Japan Institute for Health Security, Tokyo, Japan

[3] Department of Engineering Science, The University of Electro-Communications, Tokyo, Japan

[4] Research Center for Medical Sciences, The Jikei University School of Medicine, Tokyo, Japan

[5] Molecular Diagnostics and Therapeutics, The Jikei University Graduate School of Medicine, Tokyo, Japan

[6] Department of Infectious Disease Pathology, National Institute of Infectious Diseases, Japan Institute for Health Security, Tokyo, Japan

[7] Research Institute for Microbial Diseases, The University of Osaka, Osaka, Japan

[8] Graduate School of Medicine, The University of Osaka, Osaka, Japana

[9] Chulalongkorn University, Bangkok, Thailand

*Correspondence: Yuto Kotaka, kotaka.y@jihs.go.jp

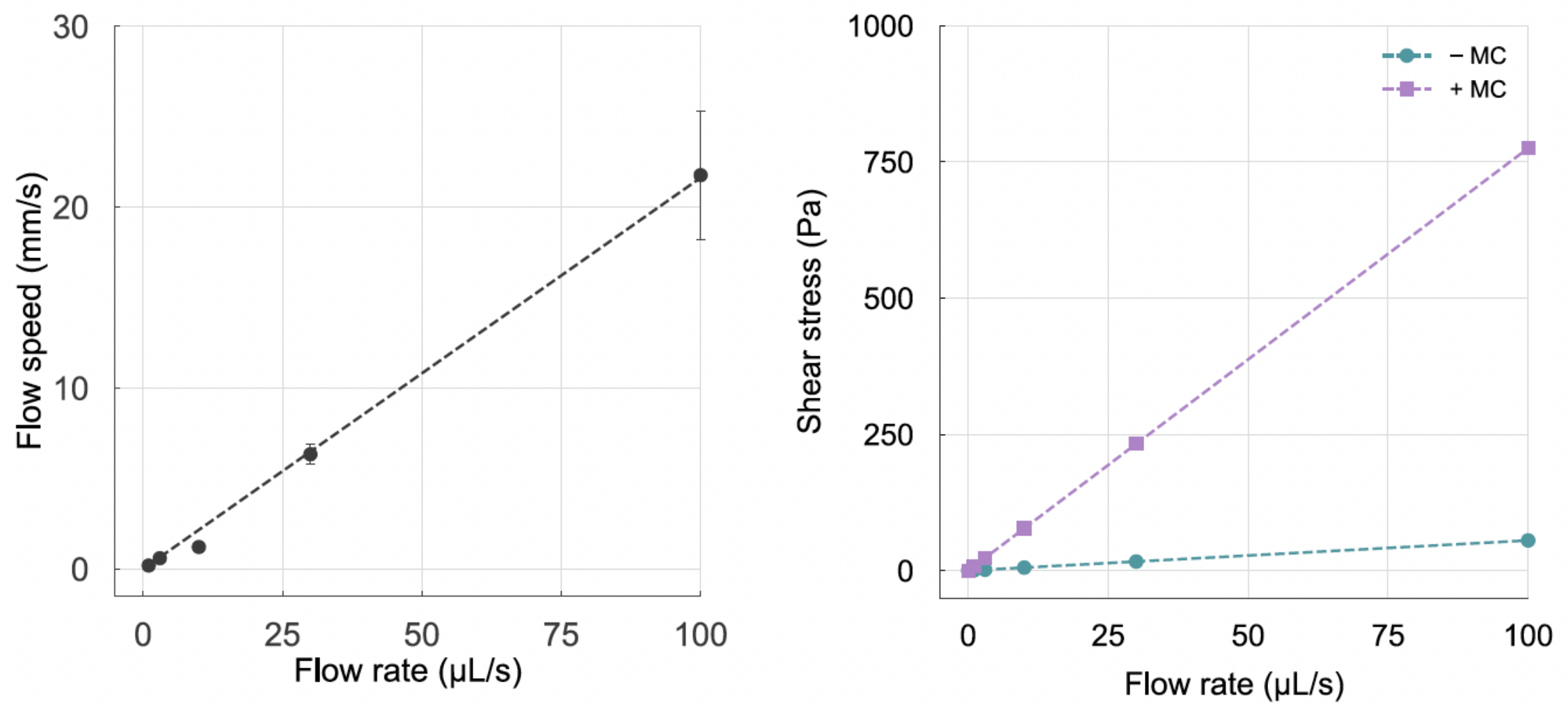

Figure S1. Flow speed characterization and estimation of shear stress

Left: Relationship between the flow rate of the syringe pump and the flow speed near the glass surface, measured from the initial velocity of broken-chain cells detached from the surface (n = 3 cells at each flow rate). Right: Relationship between the flow rate of the syringe pump and shear stress under conditions with or without MC. The shear rate, $\dot{\gamma}$, was estimated from the flow rate and the geometry of the flow channel. Shear stress was then calculated as $\sigma = \mu\dot{\gamma}$, using viscosities of 1 mPa·s for conditions without MC and 14 mPa·s for conditions with MC. Source data are provided as a Source Data file.

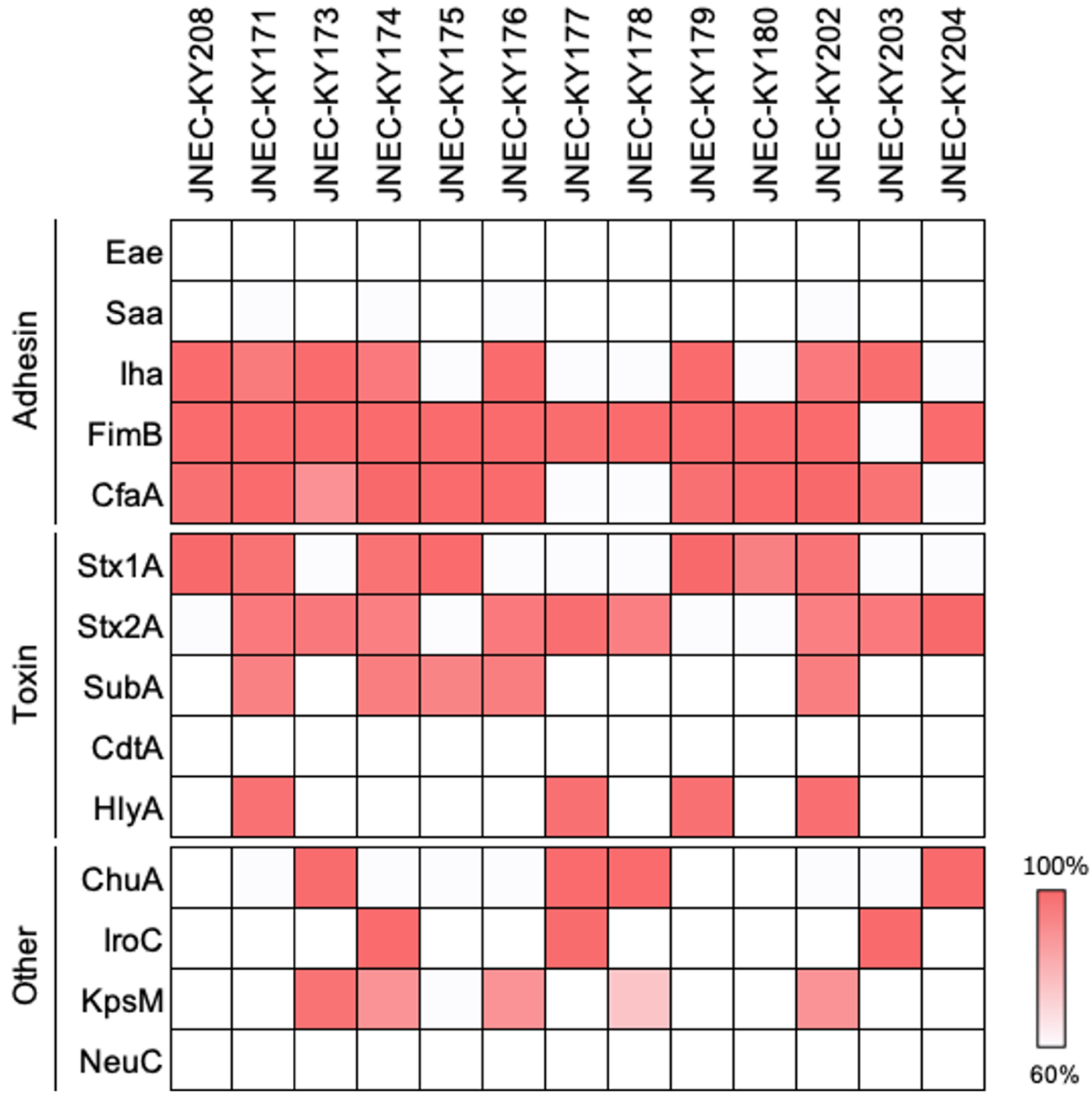

Figure S2. Virulence factor profiles of the 13 STEC isolates

A panel of virulence-associated factor proteins, including Shiga toxin and additional DEC-related factors, was screened across all isolates. Protein presence and sequence identity were assessed by BLASTp, and percent identify values were visualized as a heatmap.

```
CLUSTAL 2.1 multiple sequence alignment


ClaA            ------------------------------------------------------------
ClaB            ------------------------------------------------------------
EibD            MSKKFTMTLLSSSLAGLLVMSGG--------VSAQNGTYSVLQDDSQKSGPVKYGSTYEV
EibG            MSKKFTMTLLSSSLGGLLLGISSGVLAQELPPIKDSGLPFYSVLKEADASHVTYGSTYRI


ClaA            --------------------------------------MKNNDRKYISLLSLIPLLIAGA
ClaB            ----------------------------------------MKINRHGITLSLISFLIAGA
EibD            VKTVDNGNFRYEVKEKKNDKRTLFKFDSEGNVTVKGK--GITHTLHDPALKDFARTAEGK
EibG            YYNKSSGWGTMRYVLNGKDE-TLFNFDQEGNIIVLSKDNSIAYTVHEPVLKDFARMAAGL
                                                             :   *. :.    *

ClaA            PAFAQSDGS-------------------------------------VAYPEGNGAAASV
ClaB            PAFAQDDSS-------------------------------------WASPG--------
EibD            KNEQNGNTPPHKLTDSAVRGVYNKVYGLEKTEITGFSVED------GENGKVSLGSDAKA
EibG            RTSDKENGK-HQVDEEEVRRIFNKVNNLSNTIINPEILKQSAFIASGPRPEGDRLVQASA
                    : :

ClaA            SGNFATALGAEARATEINATAVGAHANASGRSATAVGSGTHAWSDQSTSLGTAAYVGDKA
ClaB            ----------TASASETSSTAVGAWARAG-KAGTALGSASYAHAPFSTSVGMSAYVNPKA
EibD            SGEFSVAVGNGARATEKASTAVGSWAAADGKQSTALGVGTYAYANASTALGSVAFVDNTA
EibG            AGEFALAVGTEARADKKFATSVGSWSAASGQQSVALGGGTYAYADASTALGSVAFVDNTA
                           * * :  :*:**: : *. : ..*:* .::* :  **::*  *:*. .*

ClaA            GNGMALG--SWSASDATGAVAVGTAAKAAHQNSVALGQFSGTTRENEVYIGYDSGVTKPA
ClaB            SNGVAVG--SWAKADALNSVALGTGAQATHRYSVALGQQSGTTRENEVYIGYDSGVTNPA
EibD            TYGTAAGNRAKVDKDATEGTALGAKATVTNKNSVALGANSVTTRDNEVYIGYKTGTES--
EibG            TYGTAVGNRAKVDKDATEGTALGAKATVTNKNSVALGANSRTTRDNEVYIGYEAEP----
                  * * *  :    **  ..*:*: * .::: *****  * ***:*******.:

ClaA            SPRVPDKTRVLGGVSDGTRDTDAATVGQLNRKADEVYSDVSGRIAAEALKARDHTDTVAA
ClaB            SPRVPDKTRVLGGVSDGTRDTDAATVGQLNRKADEVYSDVSGRIAAEALKARDHTDTVAA
EibD            --DKTYGTRVLGGLSDGTRNSDAATVGQLNRKVGGVYDDVKARITVESEKQKKYTDQKTS
EibG            --GKAYKTRVLGGLSDGTRPSDAATVRQVDRVKDSVEQLAQDTNTRLVVEAK--------
                    .  ******:***** :***** *::*  . * . ..   :    : :

ClaA            ENRENIIRNTVAINRNTRGLLSQRDVLETHEERLNSQQQQINTGSTVAVDSHGYVTRGEG
ClaB            ENRENIIRNTVAINRNTRGLLSQRDVLETHEERLNSQQQQINTGSTVAVDSHGYVTRGEG
EibD            -----------------------------------EVNEKVEARTTVGVDSDGKLTRAEG
EibG            -----------------------------------KSREYTDSRTTVGVNSDGTLTRAEG
                                                   . .:  :: :**.*:*.* :**.**
                         IgG Fc binding
ClaA            TGERITVQEGLVRTQEMATENRAAVS-------RNRQVGERNSRAIASQARTLQAHERRM
ClaB            TGERITVQEGLVRTQEMATENRAAVS-------RNRQVGERNSRAIASQARTLQAHERRM
EibD            ATKTIAVNDGLVALSGRTDRIDYAVGAIDGRVTRNTQSIEKNSKAIAANTRTLQQHSARL
EibG            ASKTLAVNDGLVALSGRTDRIDAAVGSVDRRVTKNTQAIQSN-------TRQLQEHNARL
                : : ::*::***  .  : .   **.       :* *  : *       :* ** *. *:

ClaA            NSQQRQINENHEEMKRAAAQSAALAGLFQPYSVGKFNATAAVGGYSDRQAVAVGMGYRFN
ClaB            NSQQRQINENHEEMKRAAAQSAALAGLFQPYSVGKFNATAAVGGYSDKQAVAVGMGYRFN
EibD            DSQQRQINENHKEMKRAAAQSAALTGLFQPYSVGKFNATAAVGGYSDQQALAVGVGYRFN
EibG            NSQQRQIRENHEEMKRAAAQSAALAGLFQPYSVGKFNATAALGGYSDKQAIAVGVGYRFN
                :******.***:************:***************:*****:**:***:*****

ClaA            EQAAAKAGTAFSDGDVSWNVGVNWEF
ClaB            EQAAAKAGTAFSDGDVSWNVGVNWEF
EibD            EQTAAKAGVAFSDGDASWNVGVNFEF
EibG            EQTAAKAGIAASDGDVSYNMGVNFEF
                **:***** * ****.*:*:***:**
```

Figure S3. Multiple sequence alignment of the newly adhesins Cla and Eib

The sequences of ClaA, ClaB, EibD, and EibG were aligned using CLUSTAL W v2.1. Residues involved in binding to IgG Fc are shown in pink.

A

```
ClaA            ------------------------------------------------------------
EibG            MSKKFTMTLLSSSLGGLLLGISSGVLAQELPPIKDSGLPFYSVLKEADASHVTYGSTYRI

ClaA            ------------------------------------------------------------
EibG            YYNKSSGWGTMRYVLNGKDETLFNFDQEGNIIVLSKDNSIAYTVHEPVLKDFARMAAGLR

ClaA            -----------MKNNDRKYISLLSLIPLLIAGAPAFAQSDGSVAYPEGNG---AAASVSG
EibG            TSDKENGKHQVDEEEVRRIFNKVNNLSNTIINPEILKQSAFIASGPRPEGDRLVQASAAG
                            ::: *: :. :. :.  * ..  : **   .: *. :*   . **.:*

ClaA            NFATALGAEARATEINATAVGAHANASGRSATAVGSGTHAWSDQSTSLGTAAYVGDKAGN
EibG            EFALAVGTEARADKKFATSVGSWSAASGQQSVALGGGTYAYADASTALGSVAFVDNTATY
                :** *:*:**** :  **:**: : ***:.:.*:*.**:*::* **:**:.*:*.:.*

ClaA            GMALG--SWSASDATGAVAVGTAAKAAHQNSVALGQFSGTTRENEVYIGYDSGVTKPASP
EibG            GTAVGNRAKVDKDATEGTALGAKATVTNKNSVALGANSRTTRDNEVYIGYEAEPGK----
                * *:*  :   .*** ..*:*: *..:::******  * ***:*******::   *

ClaA            RVPDKTRVLGGVSDGTRDTDAATVGQLNRKADEVYSDVSGRIAAEALKARDHTDTVAAEN
EibG            --AYKTRVLGGLSDGTRPSDAATVRQVDRVKDSVEQLAQDTNTRLVVEAKKSREYTDSR-
                  . *******:***** :***** *::*  *.* . ...  :  .::*:.  : . :.

ClaA            RENIIRNTVAINRNTRGLLSQRDVLETHEERLNSQQQQINTGSTVAVDSHGYVTRGEGTG
EibG            -----------------------------------------TTVGVNSDGTLTRAEGAS
                                                         :**.*:*.* :**.**:.

ClaA            ERITVQEGLVRTQEMATENRAAVSRNRQVGERNSRAIASQARTLQAHERRMNSQQRQINE
EibG            KTLAVNDGLVALSGRTDRIDAAVGSVDRRVTKNTQAIQSNTRQLQEHNARLNSQQRQIRE
                : ::*::***  .  : .  ***.    :   :*::** *::* ** *: *:*******.*

ClaA            NHEEMKRAAAQSAALAGLFQPYSVGKFNATAAVGGYSDRQAVAVGMGYRFNEQAAAKAGT
EibG            NHEEMKRAAAQSAALAGLFQPYSVGKFNATAALGGYSDKQAIAVGVGYRFNEQTAAKAGI
                ********************************:*****:**:***:*******:*****

ClaA            AFSDGDVSWNVGVNWEF
EibG            AASDGDVSYNMGVNFEF
                * ******:*:***:**
```

B

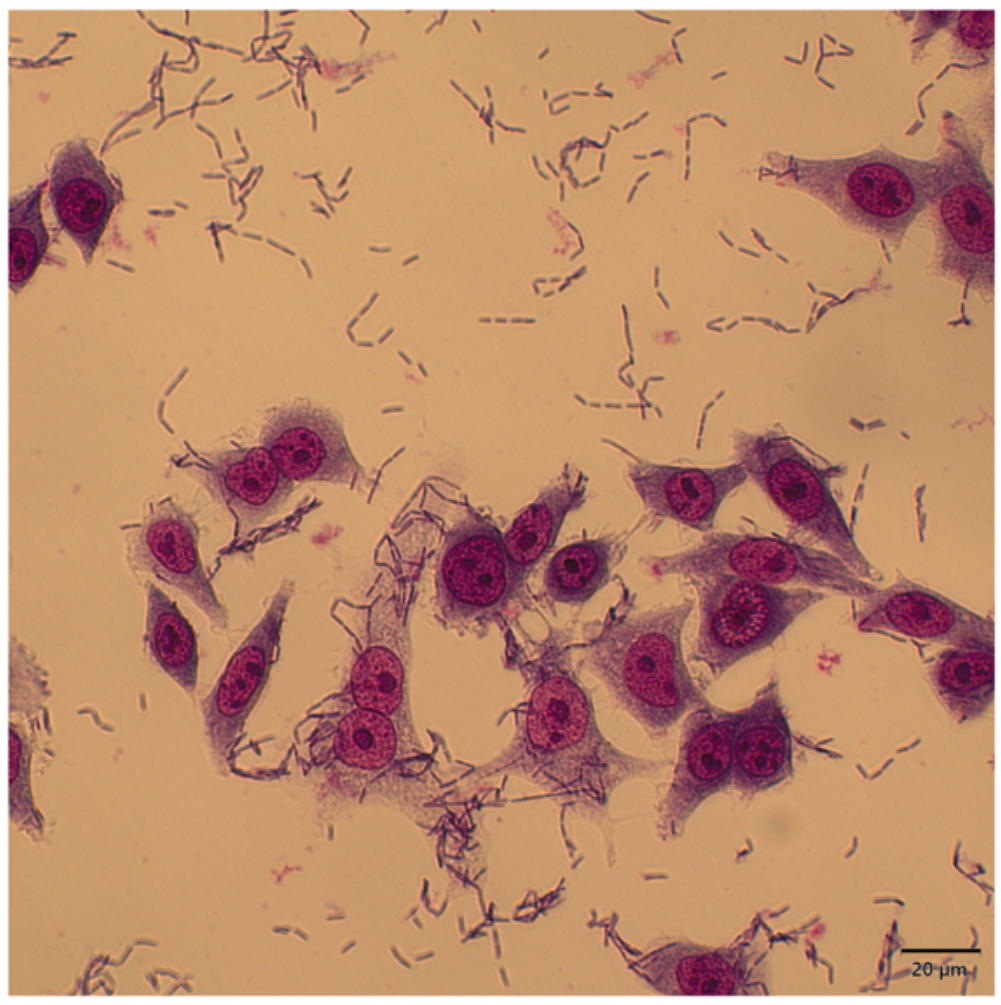

Figure S4. Construction of a chimeric EibG incorporating the IgG Fc binding region from Cla

(A) Pairwise amino acid sequence alignment between ClaA and EibG. The divergent region flanking the predicted IgG-binding domain (highlighted in pink) was substituted into the EibG scaffold to generate a chimeric construct. (B) Adhesion phenotype of HEp-2 cells infected with the chimeric Cla-type EibG. HEp-2 cells were infected at a multiplicity of infection (MOI) of 100. The chimeric protein successfully mediates the chain-like adhesion phenotype (CLAP), demonstrating that the substitution of the divergent region does not impair the structural integrity required for chain formation.

A

Strain	Year	BioSample	GenBank	Source	Serotype	Eib-like protein
ExPEC	2014	SAMN37123114	GCA_050828455.1	blood	O75:H5	HHO4750931
ExPEC	2020	SAMN37072436	GCA_044001315.1	urine	O169:H9	HFT6317502

B

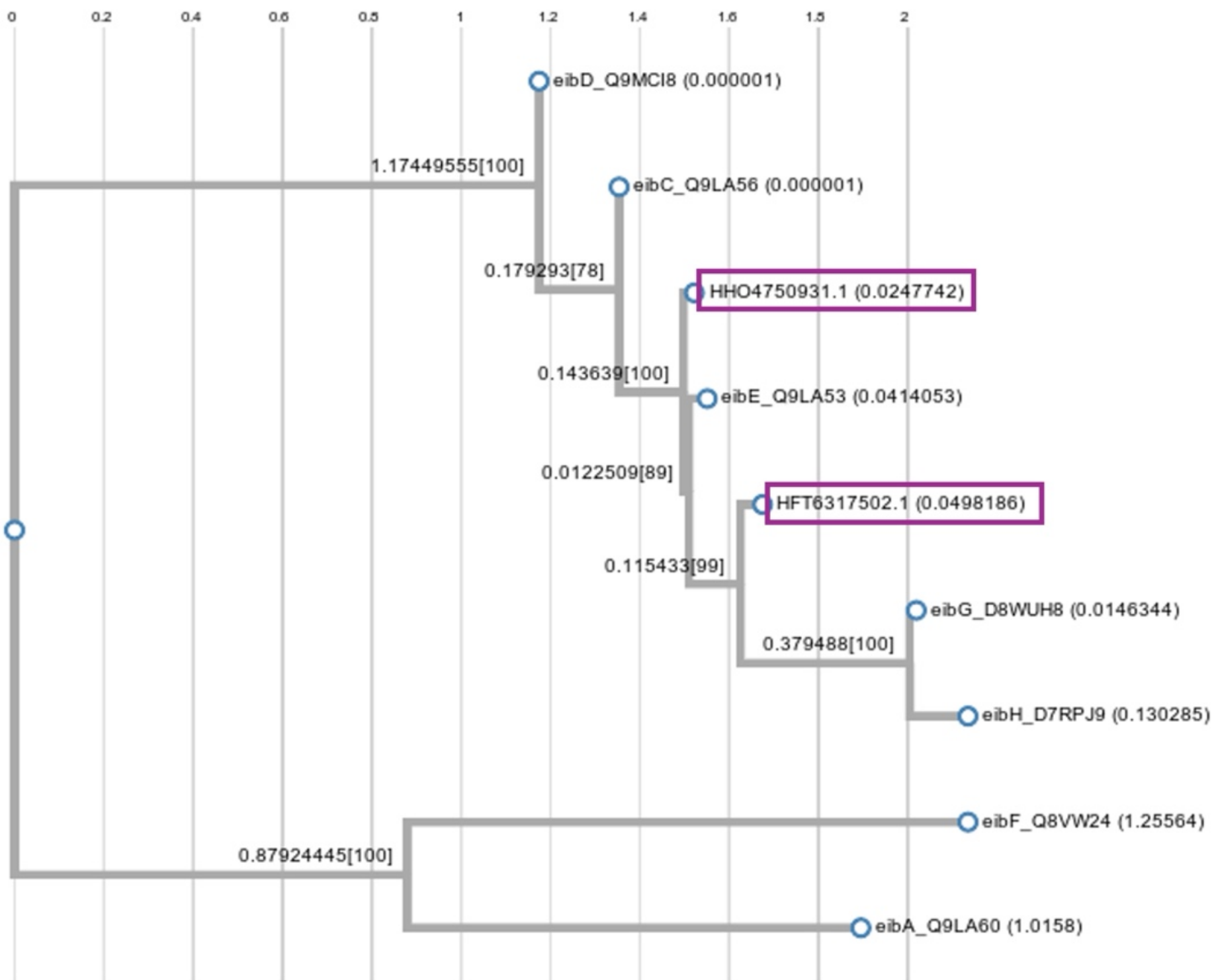

Figure S5. Eib-like proteins identified from ExPEC.

(A) Eib-like proteins screened manually from BLASTp search using EibG as the query. (B) Phylogenetic tree of Eib proteins from ExPEC. After removing signal peptide from the detected Eib-like proteins and known Eibs using SignalP6, multiple sequence alignment was performed using ClustalW 2.1, followed by phylogenetic analysis using RAxML v8.2.11 with the PROTGAMMAJTT model. Phylogenetic analysis was performed on the GenomeNet server (https://www.genome.jp).

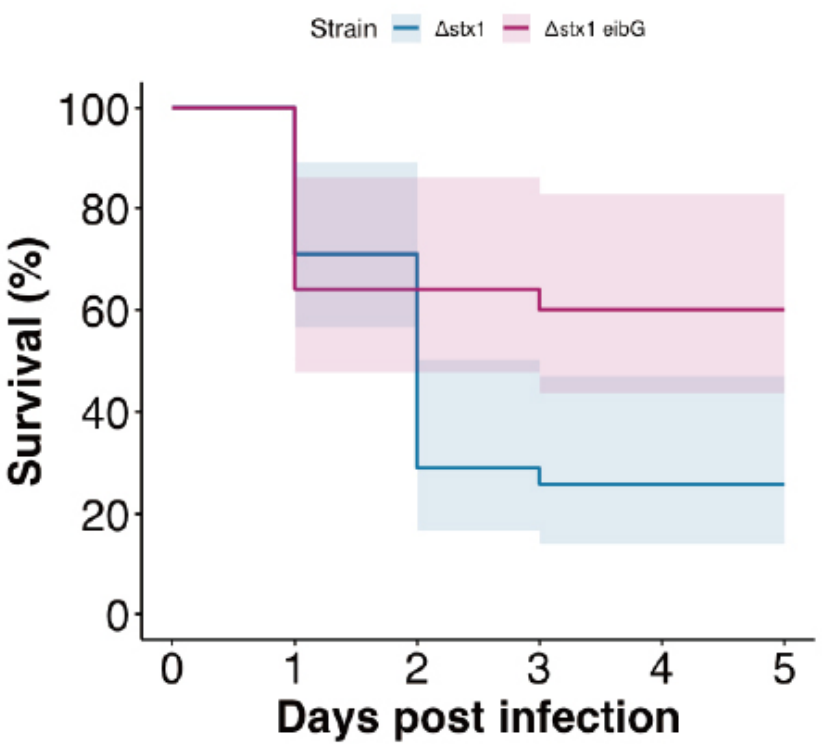

Figure S6. EibG contributes to virulence in a mouse infection model.

Survival curves of mice infected intraperitoneally with JNEC-KY208 derivatives. Specific-pathogen-free mice were inoculated intraperitoneally with 1.5×10^8 CFU of either the Δ*stx1* strain or the isogenic Δ*stx1*Δ*eibG* strain. Animals were monitored for 5 days for survival outcomes. Across three independent experiments (n = 31 for Δ*stx1*; n = 25 for Δ*stx1*Δ*eibG*), deletion of *eibG* resulted in significantly reduced lethality compared with the parental strain (log-rank test, $p = 0.044$). Shaded areas represent the 95% confidence intervals. Source data are provided as a Source Data file.
